# Refined Phylogenomics Resolves Discordance in the Aphidinae Phylogeny

**DOI:** 10.3390/genes17070755

**Published:** 2026-06-30

**Authors:** Nan Song, Xingyu Lin, Menghan He, Qingbo Tang

**Affiliations:** College of Plant Protection, Henan Agricultural University, Zhengzhou 450046, China; songnan@henau.edu.cn (N.S.); xingyulin666666@163.com (X.L.)

**Keywords:** aphid, Aphidini, Macrosiphini, genomic data, phylogeny

## Abstract

Background/Objectives: Aphids are among the most economically damaging agricultural pests globally. However, the internal phylogeny of their largest subfamily, Aphidinae, remains contentious, particularly regarding intergeneric relationships, tribal boundaries, and monophyletic status. Here, we coupled refined taxon sampling with rigorous data-filtering strategies to resolve these persistent phylogenetic discordances. Methods: We sequenced the genomes of four aphid species: *Lipaphis pseudobrassicae*, *Rhopalosiphum maidis*, *Hyalopterus arundiniformis*, and *Uroleucon helianthicola*. Integrating these new data with public genomic and transcriptomic resources, we established a comprehensive phylogenomic framework for Aphidinae. To address conflicting topologies, we compared a taxonomically broad 157-taxa dataset against a high-completeness 47-taxa core dataset. Furthermore, we evaluated the impact of intrinsic sequence properties, such as compositional heterogeneity and substitution rates, on phylogenetic conflict. Results: Our analyses demonstrate that filtering phylogenetically inconsistent loci and accounting for site heterogeneity significantly improve the topological stability of previously recalcitrant taxa. Conclusions: This study provides a refined evolutionary framework for the Aphidinae and underscores the critical importance of rigorous data curation when resolving rapid evolutionary radiations.

## 1. Introduction

Aphidinae is the most species-rich subfamily within the Aphididae, encompassing more than half of all known aphid species [1] and including some of the world’s most destructive agricultural pests [2,3]. Despite their significant economic impact as sap-suckers and viral vectors [4], the internal phylogenetic relationships of Aphidinae remain a subject of intense debate [5,6].

The tribe Macrosiphini comprises over 2358 described species across 255 genera, accounting for more than 40% of extant aphid diversity [1,7]. Historically, tribal relationships within the subfamily Aphididae have been unstable. While morphological hypotheses defined distinct boundaries between these groups, early molecular studies yielded conflicting results regarding the phylogenetic placement of Pterocommatinae, with some evidence suggesting its nested inclusion within a non-monophyletic Macrosiphini [8,9,10]. Choi et al. provided a multi-gene analysis (*EF-1α*, *cox1*, *cox2*, and *16S rRNA*) that supported the monophyly of Macrosiphini *sensu lato* and identified two distinct subgroups: Macrosiphini *sensu stricto* and a “*Pterocomma* group” [11]. Crucially, Choi et al. proposed the reassignment of several genera, including *Capitophorus*, *Pleotrichophorus*, *Liosomaphis*, and *Vesiculaphis*, to this *Pterocomma* group [11]. However, the stability of these placements remains uncertain due to limited sampling of Aphidini and the absence of critical representative taxa in subsequent studies.

Recent research suggests that the current circumscription of Macrosiphini likely encompasses multiple distinct subgroups of equal taxonomic rank, such as the *Cavariella*-*Pterocomma* complex [7]. However, resolving the phylogenetic relationships among these subgroups has proven difficult. Previous molecular efforts often struggled with low resolution, likely due to the rapid adaptive radiation characteristic of the subfamily [12,13]. Furthermore, single-gene or few-gene sequences often lack sufficient phylogenetic signal to overcome the “anomaly zone”, where gene tree discordance and incomplete lineage sorting obscure the true species tree [14]. While recent phylogenomic approaches have begun to address these issues [14], they have frequently been constrained by incomplete sampling of key Macrosiphini lineages, leaving the positions of many critical genera unresolved.

In this study, we aim to clarify the phylogenetic relationships within Aphidinae using a phylogenomic approach. We focused on the stability of the two major tribes, Aphidini and Macrosiphini, and evaluated how topological support is influenced by various data filtering strategies, such as selecting high-completeness matrices, removing internally inconsistent genes, and employing subsets with different evolutionary rates. Through refined sampling and the implementation of robust analytical pipelines, we present a more stable phylogenomic framework for this economically significant insect group.

## 2. Materials and Methods

### 2.1. Ethics Declaration

No specific permits were required for the insect specimens collected for this study in China. The specimens of 4 aphid species (*L. pseudobrassicae*, *R. maidis*, *H. arundiniformis*, and *U. helianthicola*) were collected in the fields. The field studies did not involve endangered or protected species. All sequenced insects are common species in China and are not included in the “List of Key Protected Wild Animals in China”. This list designates wildlife species protected under the Wildlife Protection Law of the People’s Republic of China and is jointly formulated by the National Forestry and Grassland Administration and the Ministry of Agriculture and Rural Affairs.

### 2.2. Sample Collection and DNA Extraction

In this study, we sequenced novel genomes from four aphid species: *L. pseudobrassicae*, *R. maidis*, *H. arundiniformis*, and *U. helianthicola*. These target taxa were selected to address key genomic gaps within the Aphidinae while representing diverse agricultural impacts in China. Among them, *L. pseudobrassicae* and *R. maidis* are highly destructive, widespread pests of brassica and major cereal crops, respectively. *H. arundiniformis* is a common host-alternating pest of stone-fruit orchards with limited existing data, whereas *U. helianthicola* is a localized specialist on Asteraceae that previously lacked any genome data. Sequencing these species simultaneously facilitates economic pest management and expands aphid genomic biodiversity.

Collection details for these specimens are as follows: *L. pseudobrassicae* was collected from Chinese cabbage (*Brassica rapa* subsp. *pekinensis*) in Zhengzhou, Henan Province (34°46′ N, 113°40′ E) in October 2021; *R. maidis* was collected from maize (*Zea mays*) in Yaoshan, Henan Province (33°47′21″ N, 112°19′40″ E) in July 2022; *H. arundiniformis* was obtained from robusta crabapple (*Malus robusta*) in Taihang Mountains, Anyang County, Henan Province (36°07′ N, 113°81′ E) in July 2021; and *U. helianthicola* was collected from sunflower (*Helianthus annuus*) in Yintiaoling, Chongqing (56°38′ N, 109°47′ E) in July 2022. Voucher specimens for *L. pseudobrassicae* (voucher number: G. Aphid_20220604), *R. maidis* (G.Aphid_20240329), *H. arundiniformis* (G. Aphid_20220606), and *U. helianthicola* (G.Aphid_20240326) were deposited at the Henan International Laboratory for Green Pest Control, Henan Agricultural University (Zhengzhou, China). Prior to DNA extraction, all specimens were preserved in absolute ethanol and stored at −80 °C.

Species were primarily identified based on morphology, supplemented by DNA barcoding molecular identification (https://id.boldsystems.org/ (accessed on 25 June 2026)). Furthermore, we performed de novo assembly of the mitochondrial genomes for the four species studied. Species identification was further validated through sequence alignment with available accessions in GenBank. Relevant data can be obtained by contacting the corresponding authors of this study.

For each species, 10–20 individuals were utilized for genomic DNA extraction. DNA was extracted from tissue using the TIANamp Genomic DNA Kit (TIANGEN BIOTECH CO., LTD., Beijing, China) following the manufacturer’s protocols. DNA quantification was conducted using the Quant-iT PicoGreen dsDNA Assay Kit, Invitrogen, Carlsbad, CA, USA, while integrity was assessed by electrophoresis on a 2% agarose gel.

### 2.3. Sequencing and Assembly

Genomic DNA was fragmented using the Covaris sonicator E220 (Covaris, Brighton, UK). The DNA library for each aphid species was prepared using the DNBSEQ DNB Rapid Prep Kit V2.0 (MGI Tech Co., Shenzhen, China), with an insert size of approximately 450 bp. Genome sequencing was performed using the MGI DNBSEQ-T7 platform (paired-end 150 bp reads). The quality of raw data was assessed using FastQC [15]. Trimmomatic v0.32 [16] was used to remove adapters, filter low-quality reads, and retain high-quality reads with a Q30 score exceeding 90% for genome assembly. Minia 3 [17] was employed for de novo assembly, utilizing a multi-kmer strategy (k-mers: 41, 61, 81, 101, and 121). Genome assembly quality statistics were computed using SeqKit [18].

### 2.4. Ortholog Assignment and Phylogenetic Matrix Construction

We used the Benchmarking Universal Single-Copy Orthologs (BUSCOs) pipeline [19] to identify universal single-copy orthologs from the genomes and transcriptomes of all species in the phylogenetic analysis, employing the Hemiptera_odb10 database. To construct the nucleotide and amino acid supermatrices, respectively, the nucleotide and amino acid sequences of BUSCO genes from each species were initially clustered. The resulting sequences were then aligned using MAFFT v7.407 [20] employing the L-INS-I algorithm. Alignments were subsequently trimmed with trimAl-1.4.1 [21] and concatenated into supermatrices using FASconCAT-G-1.04 [22]. To assess the impact of missing data on phylogenetic reconstruction, datasets with 50%, 70%, and 90% completeness were generated using PhyKIT [23,24]. This filtering ensured that each locus included in the analysis was represented by at least 50%, 70%, or 90% of the terminal species, respectively. Additionally, the third codon positions were systematically removed from the nucleotide dataset to evaluate the influence of site saturation on phylogenetic inference. Consequently, three distinct datasets were compiled for each completeness matrix type: faa (amino acid dataset), fna_nt123 (full-site nucleotide dataset), and fna_nt12 (nucleotide dataset with the third codon positions excluded). Summary statistics for each resulting supermatrix were computed using AMAS [25] (Table 1).

### 2.5. Phylogenetic Analysis

Initially, phylogenetic reconstruction was performed using 157 terminal species, including 152 Aphidinae (54 Aphidini and 98 Macrosiphini; Table S1). Outgroup selection was informed by established systematic frameworks [13,14,26], which identify Calaphidinae, Chaitophorinae, Drepanosiphinae, and Phyllaphidinae as the closest relatives to Aphidinae. Notably, Calaphidinae was excluded from the closer outgroup due to limited gene recovery (<500 BUSCOs) in available genomic resources.

To assess whether phylogenetic signal was being confounded by the high proportion of missing data in the full matrix, we strategically constructed a core dataset comprising 47 species. By applying a stringent threshold of >2000 BUSCOs (Table S2), we aimed to maximize the signal-to-noise ratio and provide a robust phylogenomic backbone. This reduced-taxon approach served as a sensitivity analysis to evaluate the consistency of the 157-species topology and to mitigate potential systematic biases that are often exacerbated by data sparsity.

Phylogenetic trees were reconstructed using three complementary inference methods: maximum likelihood (ML), coalescent species tree analysis, and Bayesian inference. The ML analyses were conducted using IQ-TREE version 2.2.15 [27]. The alignments were partitioned by loci, and the optimal substitution models and partitioning schemes were determined using ModelFinder [28]. These specific models were selected to maintain computational efficiency across our large-scale datasets. Node support values (BS) were assessed using ultrafast bootstrapping [29] with 1000 replicates.

Coalescent species tree analyses were performed on the 50% completeness datasets (faa_50, fna_50_nt12, and fna_50_nt123) using the weighted ASTRAL (wASTRAL) method implemented in ASTRAL version 5.7.1 [30,31]. ML trees for each locus alignment were generated using IQ-TREE version 2.2.15, employing the GTR model for nucleotide alignments and the LG model for amino acid alignments. The faa_50, fna_50_nt12, and fna_50_nt123 datasets were selected for species tree inference in ASTRAL because the 50% occupancy threshold provided an optimal trade-off between taxon coverage and data quantity. Compared to more stringent filters (e.g., 70% or 90% occupancy), which severely restricted the number of retained loci and total sequence length in exploratory trials, these 50% occupancy datasets maximized the number of individual loci available. Because coalescent-based summary methods rely heavily on the quantity and historical signal of underlying gene trees, retaining a larger gene pool effectively maximizes the phylogenetic signal required to resolve deep nodes while minimizing downstream coalescent errors caused by uninformative loci. Local posterior probabilities (LPPs) [32] were calculated to provide support values for the wASTRAL-inferred topologies.

Bayesian phylogenetic inference was performed using PhyloBayes-MPI v1.9 [33]. To accommodate across-site base compositional heterogeneity, we implemented the site-heterogeneous CAT–Poisson model, a non-parametric infinite mixture model that automatically estimates the number of site categories and their profiles from the data. Previous studies have demonstrated that the CAT mixture models implemented in PhyloBayes consistently outperform traditional site-homogeneous models in mitigating systematic errors during phylogenetic reconstruction [34,35,36,37,38]. Given that formal model selection using genome-scale datasets requires a prohibitive amount of computational power, we did not perform explicit model testing here; instead, the CAT–Poisson model was directly applied to the 47taxa_fna90_nt12 nucleotide dataset to investigate its capacity to improve phylogenetic resolution through subsequent comparison with homogeneous models. For this analysis, standard default uninformative priors were applied, including a uniform prior over all tree topologies, to ensure that the posterior inferences were driven objectively by the genomic data rather than pre-existing systematic biases. To optimize computational efficiency under this demanding model, constant sites were excluded from the analysis. Two independent runs, each with two parallel MCMC chains, were performed for a minimum of 10,000 iterations. Constant sites were removed to reduce computation time. We assessed convergence using the bpcomp and tracecomp programs. Convergence was considered acceptable based on two criteria: a maxdiff of <0.3 and a minimum effective size of >100. The first 1000 trees from each MCMC chain were discarded as burn-in, and a majority-rule consensus tree was generated from the remaining trees.

Incongruence between concatenation-based (IQ-TREE) and coalescent-based (wASTRAL) phylogenies is a frequent challenge in phylogenomics, often stemming from gene tree estimation error or incomplete lineage sorting. Following the framework proposed by Shen et al. [39], we sought to reconcile these topological conflicts by identifying and filtering ‘inconsistent’ loci. These are defined as loci exhibiting high-conflict signals that disproportionately destabilize the species tree. Our analysis focused on two 47-taxon datasets, specifically faa_50 and fna_50_nt12. To mitigate phylogenetic noise and refine the evolutionary signal, we identified and excluded discordant loci using a custom Bash script [40] (available at GitHub: https://github.com/xtmtd/Phylogenomics/tree/main/scripts/version2, accessed on 24 June 2026). Specifically, this script evaluated the directional consistency between the gene-wise log-likelihood score (GLS) and the gene-wise quartet score (GQS) for each locus. A gene locus was classified as “consistent” and retained if and only if its GLS and GQS shared the identical mathematical sign (i.e., both scores were >0 or both were <0). Conversely, loci exhibiting conflicting signs between the two metrics (representing contradictory phylogenetic signals) were defined as inconsistent and completely discarded. This filtering workflow resulted in a finalized dataset containing 1455 consistent loci that encompassed an aggregate of 1,790,343 aligned sites, with an average locus length of 1230.47 bp. This approach functioned as a sensitivity analysis to assess how inconsistent loci influence topological stability. By systematically removing these conflicting signals, we aimed to reconstruct a more robust and congruent phylogenetic framework while minimizing the potential for systematic errors during downstream inference.

To explore the effect of heterogeneity in evolutionary rates on tree reconstruction, we also calculated the evolutionary rate for each locus of the fna_50_nt123 dataset and sorted them from lowest to highest. First, the evolutionary rate for each individual locus was calculated, and all loci were subsequently ranked in ascending order based on their respective rates. Next, we partitioned the sorted loci into distinct, non-overlapping bins of 300 loci each to construct multiple concatenated data matrices representing different evolutionary rate spectrums (e.g., from the slowest to the fastest evolving groups). By reconstructing phylogenetic trees independently for each 300-locus matrix, we systematically evaluated how varying levels of evolutionary rate influence topological stability and nodal support within the Aphidinae.

## 3. Results

### 3.1. Genome Sequencing and Supermatrix

**Figure 1:**
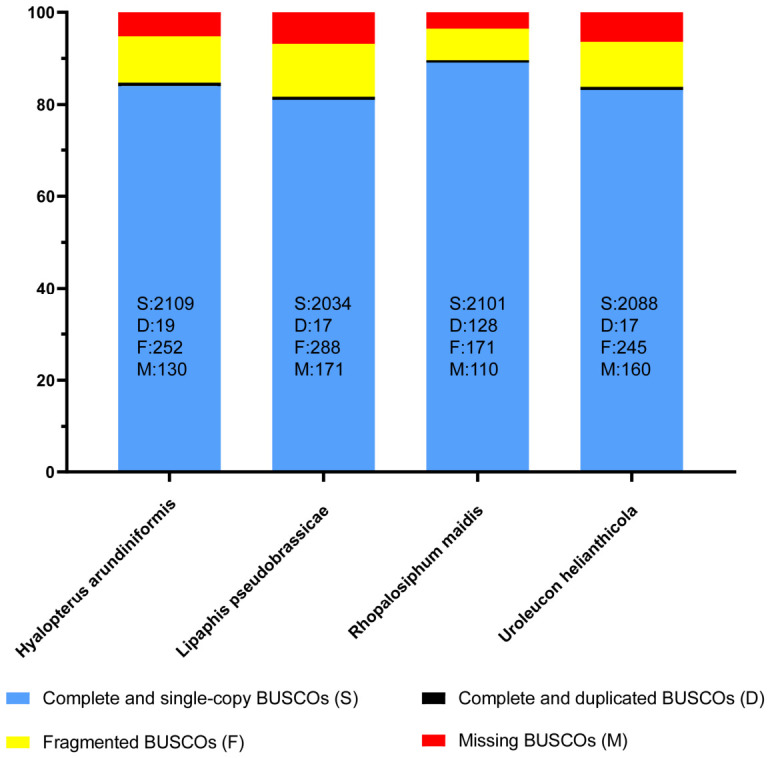
The bar charts display the proportional distribution of universal single-copy ortholog (USCO) classifications across the sequenced genomes. The categories are defined by color: complete single-copy (blue), complete duplicated (black), fragmented (yellow), and missing (red).

Comprehensive details regarding the genome sequencing and assembly metrics for the newly sequenced species are summarized in Table S3. For these four species (*L. pseudobrassicae*, *U. helianthicola*, *H. arundiniformis*, and *R. maidis*), BUSCO assessments indicated high assembly completeness, with total detection rates (comprising complete, duplicated, and fragmented genes) ranging from 93.19% to 95.96%. Specifically, the number of complete, single-copy orthologs varied between 2034 and 2109, representing 81.04% to 84.02% of the total BUSCO set (Figure 1).

Across the broader taxon sampling of 157 species used in the phylogenetic analyses, the distribution of recovered single-copy orthologs exhibited substantial variation. We categorized these species into three tiers based on gene recovery: 44 species retained a high number of loci (1642–2491), three species showed intermediate counts (771–976), and the remaining 110 species possessed a lower range of 37 to 302 genes. The detailed statistics for single-copy gene predictions across all sampled taxa are provided in Table S2.

For the 157 terminal species datasets, the supermatrix composition was inversely related to the occupancy threshold. The BUSCO50 dataset contained the most data with 190 loci, corresponding to 190,390 nucleotide sites and 63,459 amino acid sites. Increasing the occupancy to 70% (BUSCO70) reduced the data to 113 loci (105,853 nucleotide sites; 35,261 amino acid sites), and the 90% occupancy dataset (BUSCO90) yielded the most conservative matrix with only 26 loci (24,597 nucleotide sites; 8199 amino acid sites). Nucleotide datasets with the third codon position removed had fewer sites (Table 1). In contrast, both the number of loci and the total number of sites were substantially higher in the corresponding datasets assembled for the 47-species subset (Table 1).

### 3.2. Phylogenetic Inference

#### 3.2.1. Maximum Likelihood Analysis

Trees inferred from the matrices with 157 terminal species supported the subfamily Aphidinae as a monophyletic group (Figures S1–S9). Maximum likelihood analyses of the full-site nucleotide datasets consistently recovered a monophyletic Aphidini (Figures S1–S3). Conversely, the monophyly of Macrosiphini was sensitive to data filtering: it was recovered as monophyletic only in the 157taxa_fna70_nt123 dataset (Figure S2), while appearing paraphyletic in the fna50 (Figure S1) and fna90 (Figure S3) versions. Notably, amino acid-based phylogenies failed to recover the monophyly of either Aphidini or Macrosiphini (Figures S4–S6). Excluding the third codon positions yielded no improvement in resolving relationships within the subfamily (Figures S7–S9).

**Figure 2:**
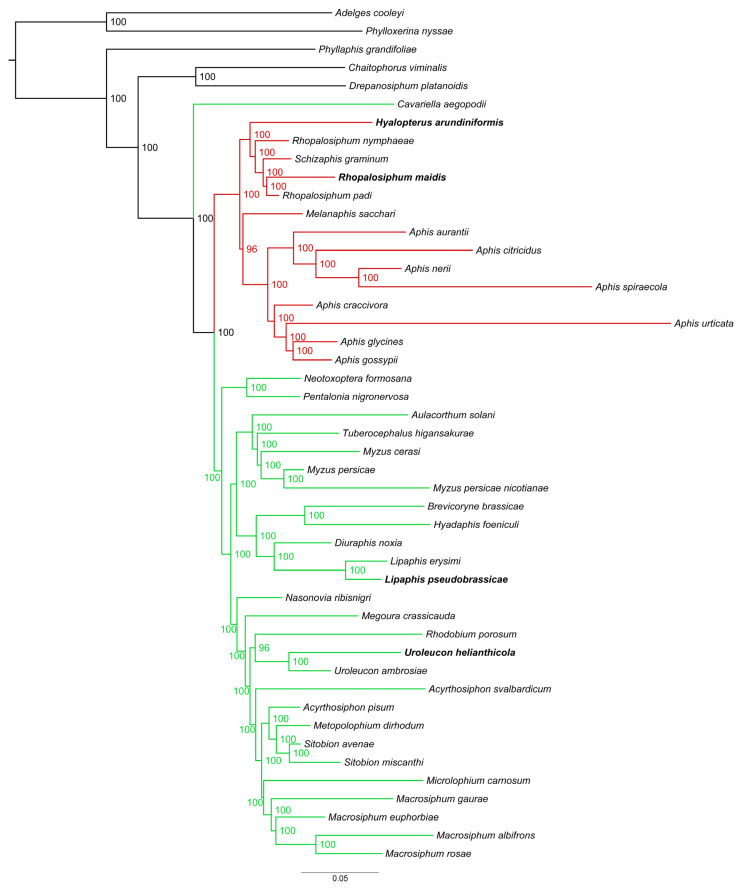
Phylogenetic relationships within the subfamily Aphidinae. Maximum likelihood trees were generated using the program IQ-TREE based on the dataset 47taxa_faa90 (561,403 amino acid positions). Nodal support is displayed as bootstrap values. Species newly sequenced for this study are highlighted in bold text. Branch colors denote tribal assignment: Macrosiphini (green) and Aphidini (red).

Phylogenetic analysis of the 47-species dataset, which had higher data completeness, improved the resolution of relationships within Aphidinae compared to the initial 157-species analysis. Specifically, phylogenetic inferences from the 47-species dataset consistently recovered the same tribal relationships within Aphidinae (Figure 2 and Figures S10–S12). In these trees, Aphidini was monophyletic, while Macrosiphini was paraphyletic, with *Cavariella aegopodii* forming a distinct and distant clade from the majority of Macrosiphini species.

#### 3.2.2. Species Tree Analysis

**Figure 3:**
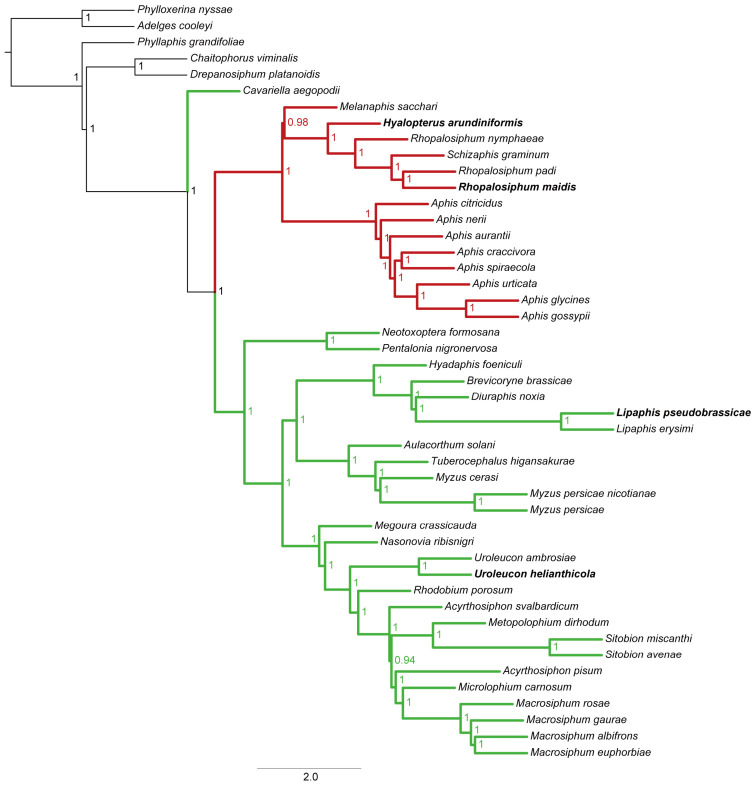
Species tree relationships within the subfamily Aphidinae. The species tree was reconstructed using the weighted ASTRAL (wASTRAL) method implemented in ASTRAL version 5.7.1. The analysis used gene trees derived from the dataset 47taxa_faa50 (1,154,453 amino acid positions). Nodal support is indicated by local posterior probabilities. Species newly sequenced for this study are highlighted in bold text. Branch colors denote tribal assignment: Macrosiphini (green) and Aphidini (red).

Species tree analyses using the wASTRAL method implemented in ASTRAL v 5.7.1. recovered a backbone topology largely congruent with the concatenated ML results. However, localized incongruences were observed regarding specific taxon placements and certain interspecific relationships (Figure 3 and Figure S13). For instance, while the three wASTRAL analyses yielded identical placements for *Megoura crassicauda* and *Nasonovia ribisnigri*, their positions shifted significantly in the concatenated ML trees. Furthermore, the phylogenetic placement of *Melanaphis sacchari* varied by data type: the nucleotide datasets (47taxa_fna50_nt12 and 47taxa_fna50_nt123) supported a sister relationship to Aphis, whereas the amino acid dataset (47taxa_faa50) resolved *M. sacchari* as sister to a clade containing *Hyalopterus*, *Rhopalosiphum*, and *Schizaphis*. To resolve these topological conflicts, we employed three additional phylogenomic strategies: Bayesian inference under the site-heterogeneous CAT–Poisson model and ML analyses involving either the exclusion of inconsistent loci or the use of gene subsets partitioned by evolutionary rate.

#### 3.2.3. Bayesian Inference

**Figure 4:**
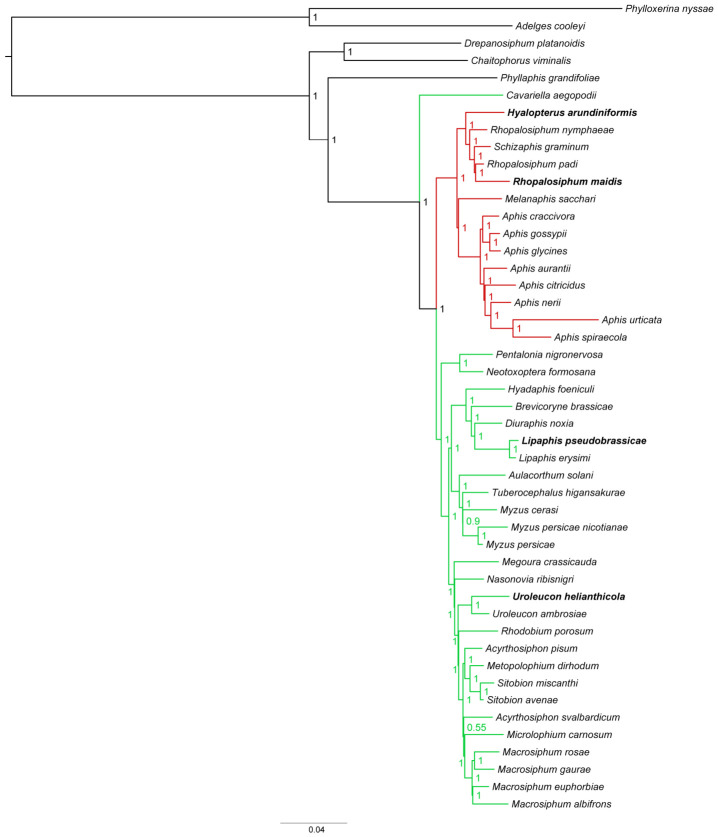
Phylogenetic relationships within the subfamily Aphidinae inferred using Bayesian analysis (CAT–Poisson). The tree was constructed with PhyloBayes-MPI from the 47taxa_fna90_nt12 dataset (1,126,611 nucleotide positions). Nodal support is shown as Bayesian posterior probabilities. Species newly sequenced for this study are highlighted in bold text. Branch colors denote tribal assignment: Macrosiphini (green) and Aphidini (red).

Bayesian analysis using the site-heterogeneous CAT–Poisson model yielded a tree topology similar to the ML analysis using the same 47taxa_fna90_nt12 dataset (Figure 4). Within the Aphidini tribe, *M. sacchari* was sister to *Aphis*. Within Macrosiphini, the placements of *M. crassicauda* and *N. ribisnigri* were consistent with the ML analysis of the 47taxa_fna90_nt123 dataset but differed from the ML analyses of both the 47taxa_fna90_nt12 nucleotide and 47taxa_faa90 amino acid datasets. The two *Acyrthosiphon* species were distantly related and fell into separate clades.

#### 3.2.4. Effects of Removing Inconsistent Genes

For the 47taxa_faa50 dataset, the initial matrix comprised 2496 loci and 1,154,453 sites. After removing inconsistent genes, we retained 1304 loci (a reduction of 1192 genes) and 547,350 sites. Similarly, for the 47taxa_fna50_nt12 dataset, the original assembly of 2496 loci containing 2,315,149 sites was filtered, resulting in a consistent subset of 1455 loci and 1,790,343 sites (with 1041 inconsistent genes removed).

The removal of inconsistent genes substantially improved topological congruence across different data types and reconstruction methods. We specifically evaluated this improvement by using the 47taxa_fna50_nt12_wASTRAL tree as a reference, as nucleotide datasets excluding the third codon positions are generally less susceptible to substitutional saturation and compositional bias. Following the exclusion of inconsistent loci, the position of *M. sacchari* was recovered as sister to the *Aphis* clade in the 47taxa_faa50_consistent_wASTRAL, 47taxa_nt12_consistent_wASTRAL, and nt12_consistent_IQTREE analyses (Figures S14 and S15). The fact that these previously discordant datasets—including the amino acid and full nucleotide partitions—converged on the same topology as the more robust nt12 reference suggests that our gene-filtering strategy successfully reduced conflicting phylogenetic noise.

However, the removal of inconsistent genes appeared to have little effect on the 47taxa_faa50_consistent_IQTREE analysis (Figure S15B), which recovered the same relationships within the Aphini group as the 47taxa_faa50_IQTREE analysis. This suggests that filtering based solely on gene consistency may not fully reconcile topological discordance in this group. Given that the Aphini group likely underwent a rapid evolutionary radiation, widespread gene tree congruence is not necessarily expected. The persistent discordance observed here may instead reflect genuine biological processes such as pervasive incomplete lineage sorting or historical introgression, combined with stochastic gene tree estimation errors stemming from limited phylogenetic signal within individual loci.

#### 3.2.5. Impact of Evolutionary Rate on Relationships

To address whether the incongruence observed in Aphidinae phylogeny stems from systematic biases associated with molecular substitution rates, we conducted a rate-stratified phylogenomic analysis. The evolutionary rates for each locus ranged from 0.4186 to 0.9584. By partitioning loci into consecutive bins of 300 genes in ascending order of their rates, we established an objective framework to monitor how phylogenetic signals and noise fluctuate as a function of sequence divergence.

Our comparative analysis revealed that the placement of six key taxa (*M. sacchari*, *H. arundiniformis*, *Brevicoryne brassicae*, *Hyadaphis foeniculi*, *M. crassicauda*, and *N. ribisnigri*) is highly sensitive to the evolutionary rate of the data utilized. Specifically, a clear topological transition was observed in the slowest-evolving loci (Bins 1–300; Figure S16), where *M. sacchari* was grouped with *Rhopalosiphum* and *Schizaphis*. This dataset also supported a sister relationship between *B. brassicae* and *H. foeniculi*, suggesting that conserved genes may retain ancestral signals that are often overwritten in faster-evolving sequences. At moderate evolutionary rates (Bins 301–600; Figure S17), a shift in topology emerged where the placement of *M. sacchari* remained stable, but the sister relationship between *B. brassicae* and *H. foeniculi* collapsed as *M. crassicauda* began to cluster with *N. ribisnigri*. At higher evolutionary rates (Bins 601–2496; Figures S18–S23), a consistent topology emerged across all datasets, placing *M. sacchari* as the sister group to the genus *Aphis*. This sister relationship reached its maximum support in the dataset containing Bins 601–900 (BS = 100, Figure S18). For the remaining high-rate datasets, bootstrap support for this node varied, ranging from 61% (Bins 1501–1800) to 97% (Bins 2101–2496).

## 4. Discussion

Initial phylogenetic analyses were performed with a high number of terminals, but they were constrained by substantial missing data, which led to inconsistent relationships across different datasets. This issue highlights a classic, widely recognized conundrum in phylogenomics: the direct trade-off between increasing taxon sampling density and maintaining matrix completeness [41,42,43]. Although comprehensive taxon sampling is essential to phylogenetic inference, missing data can introduce systematic biases [44,45,46]. Within the subfamily Aphidinae, which is characterized by ancient and rapid diversification events, such missing data can severely dilute the definitive historical signal required to resolve recalcitrant nodes. To overcome this limitation, we subsequently employed a more stringent core dataset that featured significantly less missing data and contained sufficient BUSCOs for every species’ genome. This new dataset successfully yielded a robust and well-resolved phylogeny for the subfamily Aphidinae. Despite this initial success, we still observed discordance between concatenated analyses and, more importantly, between concatenated and species tree analyses.

The reconstruction of aphid evolution represents a significant challenge in insect phylogenetics. This difficulty primarily stems from the rapid radiation of major lineages, which often leaves a weak phylogenetic signal. Furthermore, while morphological characters provide essential diagnostic information, they can exhibit considerable plasticity or convergence. These evolutionary complexities have historically led to discrepancies between traditional taxonomic frameworks and recent molecular evidence, reflecting the difficulty of reconciling different data types across various ranks in aphid systematics. To resolve these conflicting results and address potential systematic errors, we implemented a multifaceted strategy: rerunning tree reconstructions with improved inference methods and refining the datasets. The improved methods involved using Bayesian inference with a model that explicitly accounts for compositional heterogeneity and ML analysis and/or wASTRAL analyses using dataset refinement, which included removing inconsistent genes and analyzing subsets with varying evolutionary rates. Crucially, all three analyses converged on a consistent phylogenetic placement for the species that exhibited variable positions in the initial analyses, which were run with a homogeneous model and without data refinement. This consistent result specifically supports the *M. sacchari* + *Aphis* clade sister-group relationship.

### 4.1. Impact of Data Quality and Analytical Strategies on Phylogenetic Stability

In this study, the 157-taxon matrix includes several species with relatively low BUSCO recovery, resulting in an uneven distribution of missing data across the alignment. Previous simulation and empirical studies have demonstrated that high levels of uneven data completeness can decrease phylogenetic accuracy, reduce nodal support, or exacerbate systematic errors such as long-branch attraction [43,47].

Our findings indicate that data quality and pipeline selection are critical factors influencing phylogenetic stability in large-scale phylogenomic datasets. For instance, the localized topological instability observed in the 157-taxon dataset, such as the conflicting monophyly/paraphyly status of Macrosiphini across different filtering thresholds (Figures S1–S3), likely stems from an imbalance between taxon sampling and matrix completeness. In this expanded dataset, substantial missing data combined with the highly conserved nature of BUSCO loci may have diluted the informative phylogenetic signals required to resolve rapid divergence events. Conversely, the enhanced resolution and topological stability observed in the 47-taxon dataset likely reflect the synergistic effects of increased matrix completeness and reduced taxon sampling. On one hand, filtering out taxa with low data occupancy minimizes missing-data-driven artifacts and strengthens the core evolutionary signal. On the other hand, a more restricted taxon sampling reduces the complexity of the tree space and eliminates unstable lineages that cause localized conflicts in the 157-taxon tree. Ultimately, this contrast highlights the critical trade-off and complementary nature between taxon density and data completeness in phylogenomic inference.

We propose that the alignment of previously discordant datasets, such as amino acid and nucleotide matrices, toward a consistent topology following the removal of inconsistent genes provides a compelling internal line of evidence. This transition from conflict to congruence suggests that the resulting tree reflects a robust evolutionary signal rather than systematic bias. Because different data types are vulnerable to distinct systematic errors, their convergence on a single topology provides robust support for the inferred relationships. This congruence serves as an objective criterion: when independent data types (i.e., faa, fna_nt123, and fna_nt12) yield identical results only after rigorous filtering, it demonstrates that the filtering process successfully extracted the shared historical signal from conflicting noise.

Our rate-stratified analysis reveals a clear topological transition across the evolutionary rate spectrum, highlighting that the Aphidinae phylogeny is highly susceptible to rate-dependent systematic biases. The observed discrepancy—where the slowest-evolving loci (Bins 1–300) favor an alternative arrangement for *M. sacchari*, while higher-rate bins (Bins 601–2496) consistently support its sister relationship with *Aphis*—underscores the importance of locus selection. This trend suggests that highly conserved genes may lack sufficient informative sites to resolve these deep nodes, whereas the stable recovery of the *M. sacchari* + *Aphis* clade at higher rates indicates that increased sequence divergence is necessary to overcome phylogenetic noise. However, the fluctuation in bootstrap support at the highest rates (e.g., the dip to 61% in Bins 1501–1800) warns of potential mutational saturation in the most rapidly evolving sequences. Our results provide a practical benchmark for data filtering, suggesting that loci with moderate to high–moderate evolutionary rates (0.749–0.786, corresponding to genes in Bins 601–900) strike an optimal balance between signal and noise for Aphidinae. These loci retain sufficient historical signal to resolve rapid radiations while remaining resistant to the phylogenetic artifacts that typically plague extreme rate categories. This framework offers an objective standard for future studies to prioritize data that maximize phylogenetic resolution while minimizing systematic error.

While the removal of taxa (from 157 to 47) might seem to limit the breadth of the study, the resulting increase in matrix completeness and the subsequent topological stability allow for a more confident resolution of key genera. The stable recovery of the *M. sacchari* + *Aphis* clade, corroborated by a site-heterogeneous model and consistent-gene filtering, offers a refined phylogenetic framework. The reliability of evolutionary inferences is fundamentally validated when independent datasets and diverse analytical methods yield congruent results [48,49]. Consequently, these strategies effectively mitigate systematic errors prevalent in aphids, ensuring that the inferred relationships reflect evolutionary history rather than analytical artifacts.

### 4.2. Phylogenetic Relationships Within Aphidinae

Recent phylogenomic investigations, such as those by Owen and Miller [14], have highlighted that relationships within Aphididae are confounded by gene tree discordance, introgression, and the presence of “anomaly zone” branch lengths along the backbone. Notably, their study identified a significant introgression event between *Aphis craccivora* and *Aphis gossypii*, a discovery that introduces new complexities to aphid molecular taxonomy. Despite these potential localized reticulations, our results consistently recover *Aphis* as a monophyletic group, thereby reinforcing the findings of Owen and Miller [14]. However, we also noted that a prior study combining UCE and *COI* data recovered a non-monophyletic Aphis due to the nesting of *Braggia*, *Toxoptera*, *Brachyunguis*, *Sanbornia*, and *Siphonatrophia* [6]. Expanded taxon sampling is needed to test the monophyly of this genus.

The Aphidini tribe was divided into two primary, well-supported lineages. By incorporating additional representative taxa within this tribe, our wASTRAL analyses based on the 47taxa_fna50_nt123 and nt12 datasets further resolve the internal structure of these clades. Specifically, one major lineage was recovered, comprising the monophyletic genera *Aphis* and *M. sacchari*. The inclusion of these additional Aphidini species, while not expanding generic representation, provides further clarity on the interspecific relationships and the phylogenetic stability of these subtribes. The other major clade comprises *H. arundiniformis* + (*Rhopalosiphum nymphaeae* + (*Schizaphis graminum* + (*Rhopalosiphum padi* + *R. maidis*))). These relationships are broadly congruent with the findings of Hardy et al. [6] and Ding et al. [50]. Although the sister-group relationship between *R. maidis* and *R. padi* is consistent with Owen and Miller [14], the distant position of *R. nymphaeae* renders *Rhopalosiphum* non-monophyletic. In fact, all our analyses (including preliminary trials with broader taxon sampling) consistently support the non-monophyly of *Rhopalosiphum*. A previous study recovered *S. graminum* as sister to *R. maidis*, both of which are sister to *R. padi* [10]. Future research will require more extensive sampling and additional character data to further verify the monophyly of *Rhopalosiphum*.

In our analysis, *Acyrthosiphon* was consistently recovered as a non-monophyletic group. Similarly, the monophyly of *Acyrthosiphon* has been questioned in previous studies. For example, the ML concatenated phylogeny from Owen and Miller [14] resolved *Acyrthosiphon pisum* and *Macrosiphum euphorbiae* as sister groups, thereby rendering the genus *Acyrthosiphon* paraphyletic. Both studies have limited sampling, and similarly, more extensive sampling is needed to verify the monophyly of *Acyrthosiphon*. Besides *Rhopalosiphum* and *Acyrthosiphon*, all other genera with more than two sampled species in our analysis were monophyletic, including *Aphis*, *Myzus*, *Lipaphis*, *Uroleucon*, *Sitobion*, and *Macrosiphum*.

Within the Aphidinae, the Macrosiphini tribe was recovered as non-monophyletic across all analyses based on the 47-taxa datasets. This topological pattern is primarily driven by the placement of *C*. *aegopodii*, which consistently emerged as a distinct lineage separate from the core Macrosiphini clade. While some earlier molecular studies [11] suggested monophyly, our findings align with a growing body of evidence indicating that the tribe may be non-monophyletic. For instance, von Dohlen et al. [10] utilized multiple molecular markers (*EF-1α*, *tRNA-Leu*, and *COII*) and found *Pterocomma* nested within Macrosiphini as a sister to *Cavariella*, a result that mirrors the instability observed in our study. Similarly, Nováková et al. found evidence for the paraphyly of Macrosiphini relative to Pterocommatini using *Buchnera* endosymbiont sequences [8]. Moreover, a recent phylogenomic analysis by Jousselin et al. further corroborated this non-monophyletic arrangement [12]. Nevertheless, because the current recovery of non-monophyly hinges on the placement of a single representative (*C*. *aegopodii*) sourced from external databases, this interpretation should be treated with caution. The systematic status of Macrosiphini remains a contentious issue in aphid taxonomy. Further phylogenomic investigations with broader generic representation, including the *Cavariella* and *Pterocomma* lineages, are essential to definitively resolve these relationships.

## Figures and Tables

**Table 1:** Summary statistics for each resulting supermatrix.

Alignment_Name	No_Taxa	No_Loci	Occupancy (%)	Alignment_Length	Total_Matrix_Cells	Undetermined_Characters	Missing_Percent (%)
157taxa_faa50	157	190	50	63,459	9,963,063	4,024,193	40.39
157taxa_faa70	157	113	70	35,261	5,535,977	1,594,664	28.81
157taxa_faa90	157	26	90	8199	1,287,243	229,941	17.86
157taxa_fna50_nt123	157	190	50	190,390	29,891,230	12,069,973	40.38
157taxa_fna50_nt12	157	190	50	126,933	19,928,481	2,651,572	40.38
157taxa_fna70_nt123	157	113	70	105,853	16,618,921	4,788,397	28.81
157taxa_fna70_nt12	157	113	70	70,571	11,079,647	1,362,352	28.81
157taxa_fna90_nt123	157	26	90	24,597	3,861,729	689,802	17.86
157taxa_fna90_nt12	157	26	90	16,398	2,574,486	295,550	17.86
47taxa_faa50	47	2496	50	1,154,453	54,259,291	11,308,433	20.84
47taxa_faa70	47	2368	70	1,097,769	51,595,143	10,169,709	19.71
47taxa_faa90	47	1163	90	561,403	26,385,941	4,104,659	15.56
47taxa_fna50_nt123	47	2496	50	3,472,661	163,215,067	34,059,706	20.87
47taxa_fna50_nt12	47	2496	50	2,315,149	108,812,003	22,707,290	20.87
47taxa_fna70_nt123	47	2368	70	3,301,513	155,171,111	30,620,429	19.737
47taxa_fna70_nt12	47	2368	70	2,201,047	103,449,209	20,414,383	19.737
47taxa_fna90_nt123	47	1163	90	1,689,890	79,424,830	12,381,006	15.59
47taxa_fna90_nt12	47	1163	90	1,126,611	52,950,717	8,254,398	15.59

## Data Availability

The newly sequenced raw reads have been deposited in the NCBI Sequence Read Archive (BioProject ID: PRJNA1135342), and the relevant numbers of all data in this study are listed in Table S1.

## Supplementary Materials

The following supporting information can be downloaded at: https://www.mdpi.com/article/10.3390/genes17070755/s1, Figure S1: Maximum likelihood tree inferred from the 157taxa_fna50_nt123 dataset using IQ-TREE. Bootstrap values are displayed as nodal support. Branch colors denote tribal assignment: Macrosiphini (green) and Aphidini (red); Figure S2: Maximum likelihood tree inferred from the 157taxa_fna70_nt123 dataset using IQ-TREE. Bootstrap values are displayed as nodal support. Branch colors denote tribal assignment: Macrosiphini (green) and Aphidini (red); Figure S3: Maximum likelihood tree inferred from the 157taxa_fna90_nt123 dataset using IQ-TREE. Bootstrap values are displayed as nodal support. Branch colors denote tribal assignment: Macrosiphini (green) and Aphidini (red); Figure S4: Maximum likelihood tree inferred from the 157taxa_faa50 dataset using IQ-TREE. Bootstrap values are displayed as nodal support. Branch colors denote tribal assignment: Macrosiphini (green) and Aphidini (red); Figure S5: Maximum likelihood tree inferred from the 157taxa_faa70 dataset using IQ-TREE. Bootstrap values are displayed as nodal support. Branch colors denote tribal assignment: Macrosiphini (green) and Aphidini (red); Figure S6: Maximum likelihood tree inferred from the 157taxa_faa90 dataset using IQ-TREE. Bootstrap values are displayed as nodal support. Branch colors denote tribal assignment: Macrosiphini (green) and Aphidini (red); Figure S7: Maximum likelihood tree inferred from the 157taxa_fna50_nt12 dataset using IQ-TREE. Bootstrap values are displayed as nodal support. Branch colors denote tribal assignment: Macrosiphini (green) and Aphidini (red); Figure S8: Maximum likelihood tree inferred from the 157taxa_fna70_nt12 dataset using IQ-TREE. Bootstrap values are displayed as nodal support. Branch colors denote tribal assignment: Macrosiphini (green) and Aphidini (red); Figure S9: Maximum likelihood tree inferred from the 157taxa_fna90_nt12 dataset using IQ-TREE. Bootstrap values are displayed as nodal support. Branch colors denote tribal assignment: Macrosiphini (green) and Aphidini (red); Figure S10: Maximum likelihood trees inferred using IQ-TREE from two separate datasets: (A) 47taxa_fna90_nt12 and (B) 47taxa_fna90_nt123. Bootstrap values are displayed as nodal support. Branch colors denote tribal assignment: Macrosiphini (green) and Aphidini (red); Figure S11: Maximum likelihood trees inferred using IQ-TREE from three separate datasets: (A) 47taxa_faa50, (B) 47taxa_fna50_nt12, and (C) 47taxa_fna50_nt123. Bootstrap values are displayed as nodal support. Branch colors denote tribal assignment: Macrosiphini (green) and Aphidini (red); Figure S12: Maximum likelihood trees inferred using IQ-TREE from three separate datasets: (A) 47taxa_faa70, (B) 47taxa_fna70_nt12, and (C) 47taxa_fna70_nt123. Bootstrap values are displayed as nodal support. Branch colors denote tribal assignment: Macrosiphini (green) and Aphidini (red); Figure S13: Species tree relationships within the subfamily Aphidinae. The species tree was reconstructed using the weighted ASTRAL (wASTRAL) method implemented in ASTRAL version 5.7.1. The analysis used gene trees derived from two separate datasets: (A) 47taxa_fna50_nt12 and (B) 47taxa_fna50_nt123. Nodal support is indicated by local posterior probabilities. Species newly sequenced for this study are highlighted in bold text. Branch colors denote tribal assignment: Macrosiphini (green) and Aphidini (red); Figure S14: Phylogenetic trees inferred after removing inconsistent genes from the 47taxa_faa50 dataset. (A) Species tree inferred using the weighted ASTRAL (wASTRAL) method. (B) Maximum likelihood tree inferred using IQ-TREE. Branch colors denote tribal assignment: Macrosiphini (green) and Aphidini (red); Figure S15: Phylogenetic trees inferred after removing inconsistent genes from the 47taxa_fna50_nt12 dataset. (A) Species tree inferred using the weighted ASTRAL (wASTRAL) method. (B) Maximum likelihood tree inferred using IQ-TREE. Branch colors denote tribal assignment: Macrosiphini (green) and Aphidini (red); Figure S16: Maximum likelihood tree for the lowest evolutionary rate loci (1–300) of the 47taxa_fna50_nt123 dataset, inferred using IQ-TREE. Nodal support is indicated by bootstrap values. Branch colors denote tribal assignment: Macrosiphini (green) and Aphidini (red); Figure S17: Maximum likelihood tree for the moderately increasing evolution rate loci (301–600) of the 47taxa_fna50_nt123 dataset, inferred using IQ-TREE. Nodal support is indicated by bootstrap values. Branch colors denote tribal assignment: Macrosiphini (green) and Aphidini (red); Figure S18: Maximum likelihood tree for the subset of the 47taxa_fna50_nt123 dataset containing loci with a higher evolutionary rate (loci 601–900), inferred using IQ-TREE. Nodal support is indicated by bootstrap values. Branch colors denote tribal assignment: Macrosiphini (green) and Aphidini (red); Figure S19: Maximum likelihood tree for the subset of the 47taxa_fna50_nt123 dataset containing loci with a higher evolutionary rate (loci 901–1200), inferred using IQ-TREE. Nodal support is indicated by bootstrap values. Branch colors denote tribal assignment: Macrosiphini (green) and Aphidini (red); Figure S20: Maximum likelihood tree for the subset of the 47taxa_fna50_nt123 dataset containing loci with a higher evolutionary rate (1201–1500), inferred using IQ-TREE. Nodal support is indicated by bootstrap values. Branch colors denote tribal assignment: Macrosiphini (green) and Aphidini (red); Figure S21: Maximum likelihood tree for the subset of the 47taxa_fna50_nt123 dataset containing loci with a higher evolutionary rate (1501–1800), inferred using IQ-TREE. Nodal support is indicated by bootstrap values. Branch colors denote tribal assignment: Macrosiphini (green) and Aphidini (red); Figure S22: Maximum likelihood tree for the subset of the 47taxa_fna50_nt123 dataset containing loci with a higher evolutionary rate (1801–2100), inferred using IQ-TREE. Nodal support is indicated by bootstrap values. Branch colors denote tribal assignment: Macrosiphini (green) and Aphidini (red); Figure S23: Maximum likelihood tree for the subset of the 47taxa_fna50_nt123 dataset containing loci with the highest evolutionary rate (loci 2101–2496), inferred using IQ-TREE. Nodal support is indicated by bootstrap values. Branch colors denote tribal assignment: Macrosiphini (green) and Aphidini (red); Table S1: Taxa included in this study, along with corresponding accession numbers [51,52,53,54,55,56,57,58,59,60,61]; Table S2: Complete BUSCO statistics for taxa in this study; Table S3: Statistics for the new genome sequencing and assembly.
