# Supplementary figures and images for: Refined Phylogenomics Resolves Discordance in the Aphidinae Phylogeny
Source: Genes (Basel). 2026 Jun 30;17(7):755. doi: 10.3390/genes17070755 (PMC13409620; doi:10.3390/genes17070755)

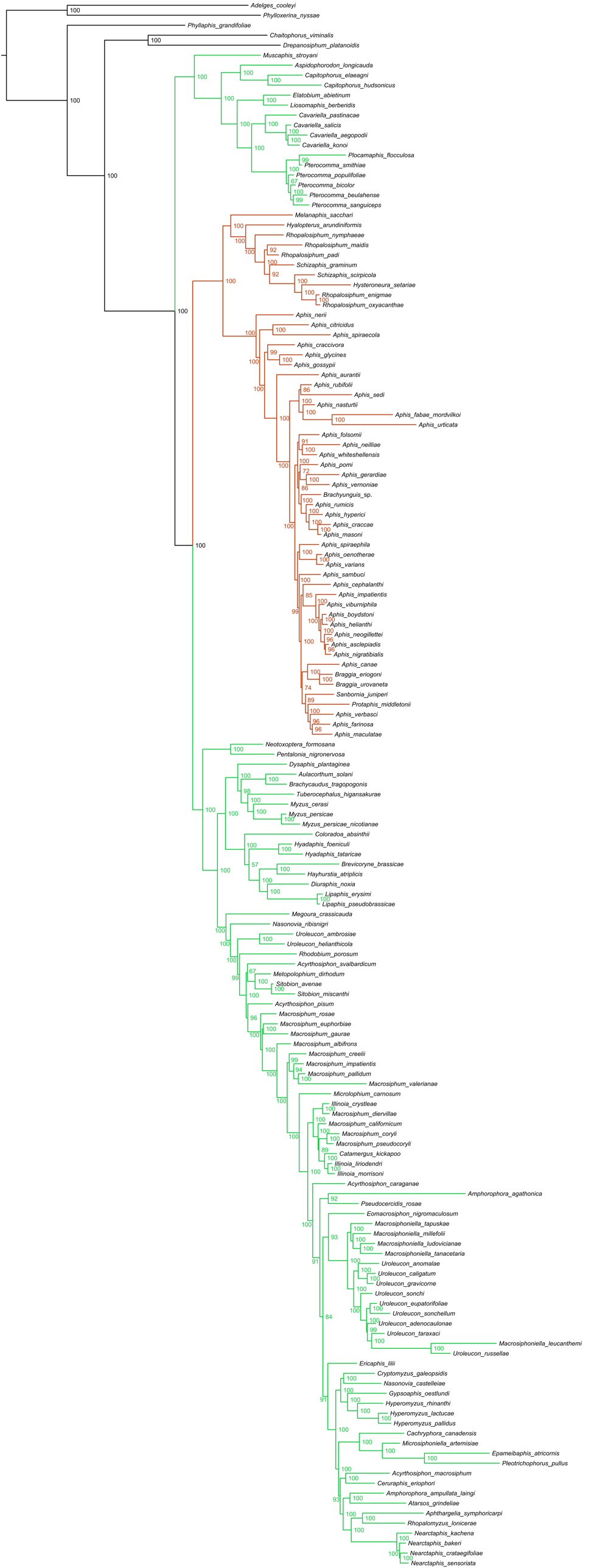

Supplement: Supplementary file 1 [file genes-17-00755-s001.zip › Figure_S1.pdf]

(A) 47taxa fna90 nt12

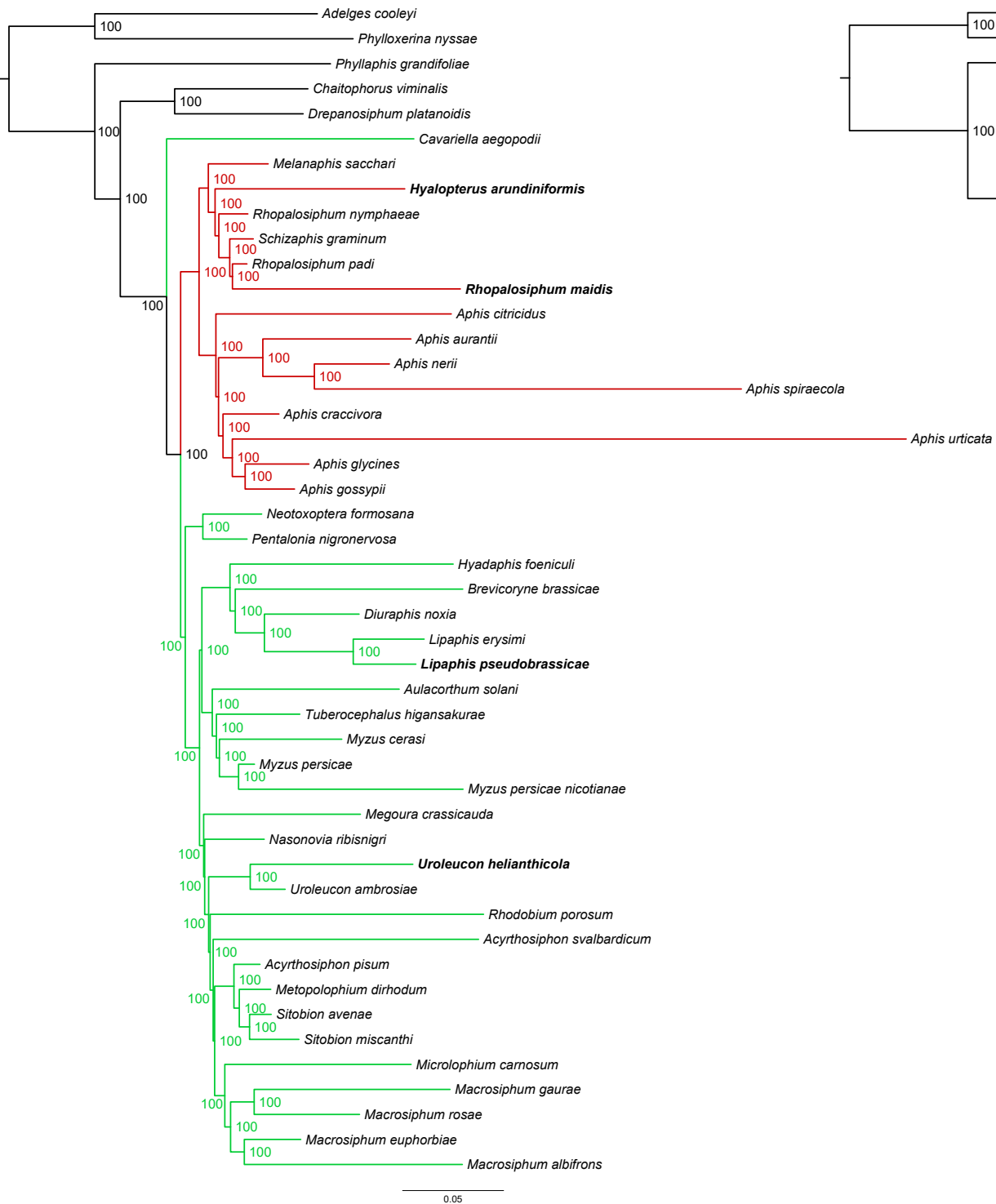

(B) 47taxa fna90 nt123

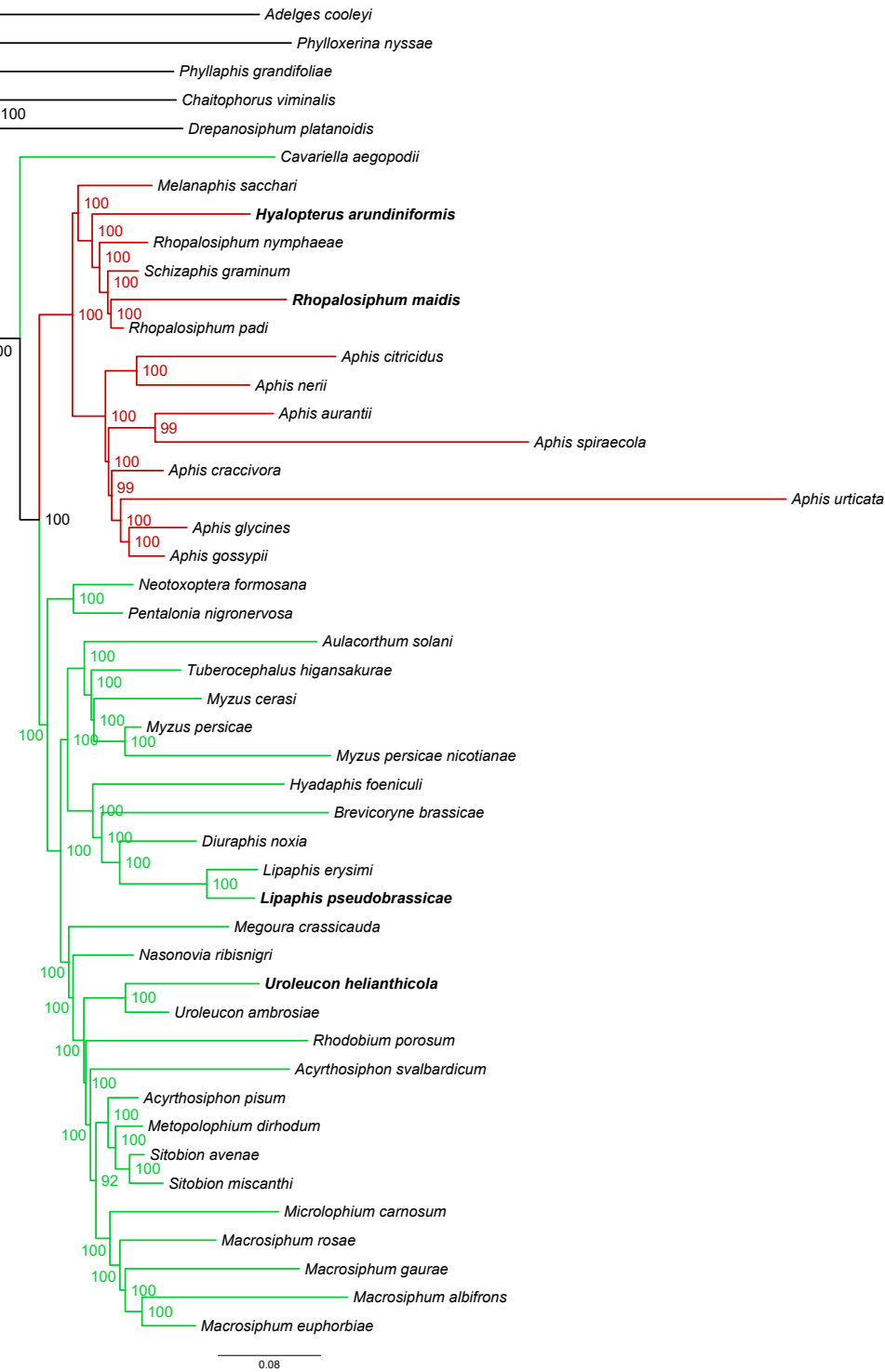

Supplement: Supplementary file 1 [file genes-17-00755-s001.zip › Figure_S10.pdf]

(A) 47taxa\_faa50

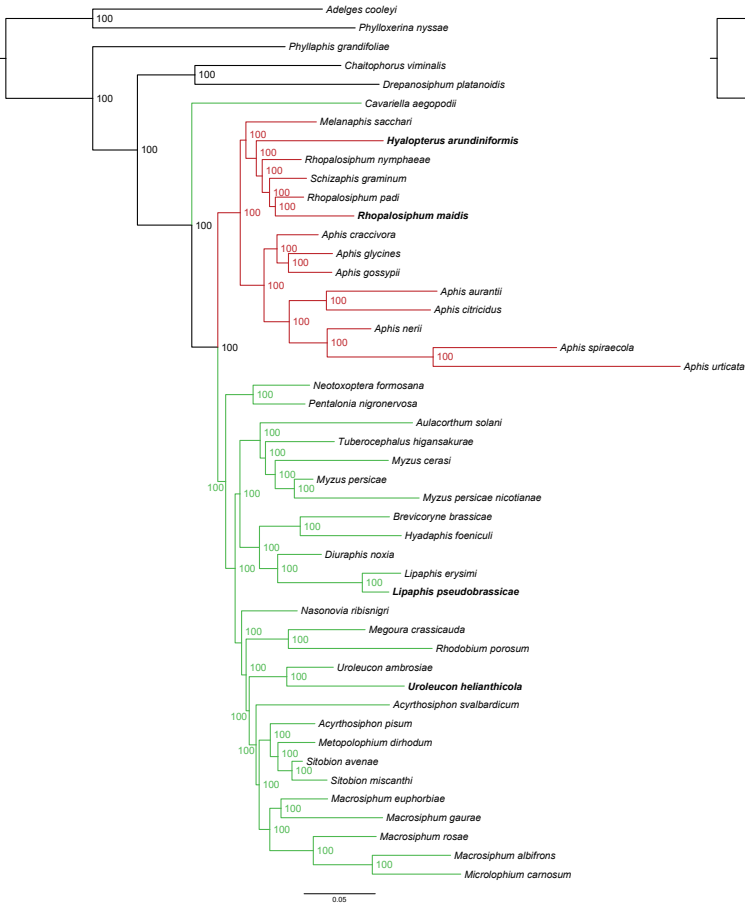

(B) 47taxa\_fna50\_nt12

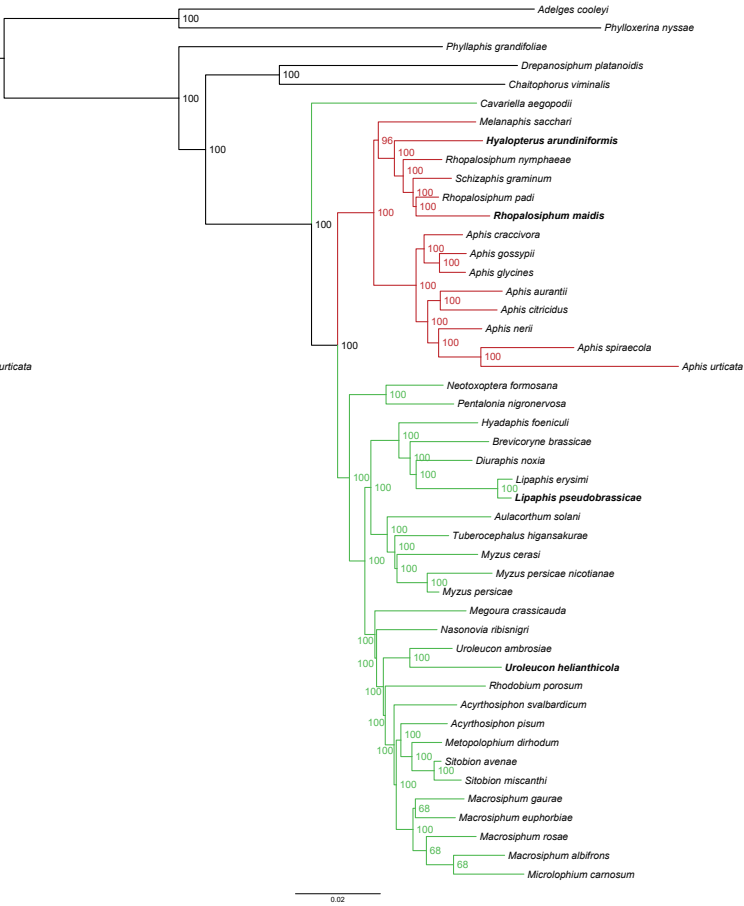

(C) 47taxa\_fna50\_nt123

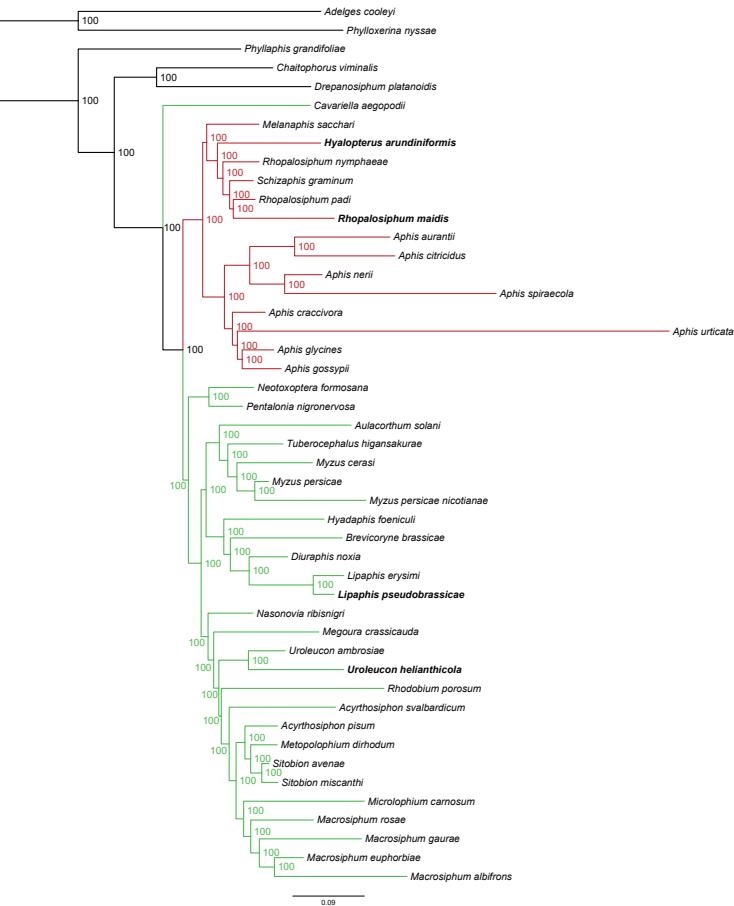

Supplement: Supplementary file 1 [file genes-17-00755-s001.zip › Figure_S11.pdf]

(A) 47taxa\_faa70

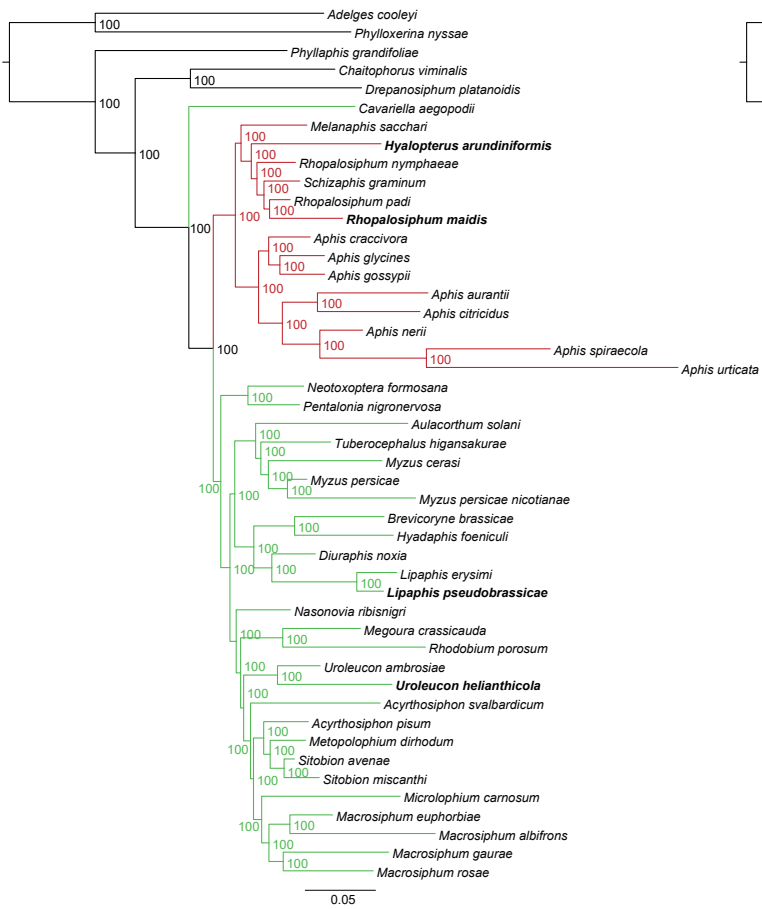

(B) 47taxa\_fna70\_nt12

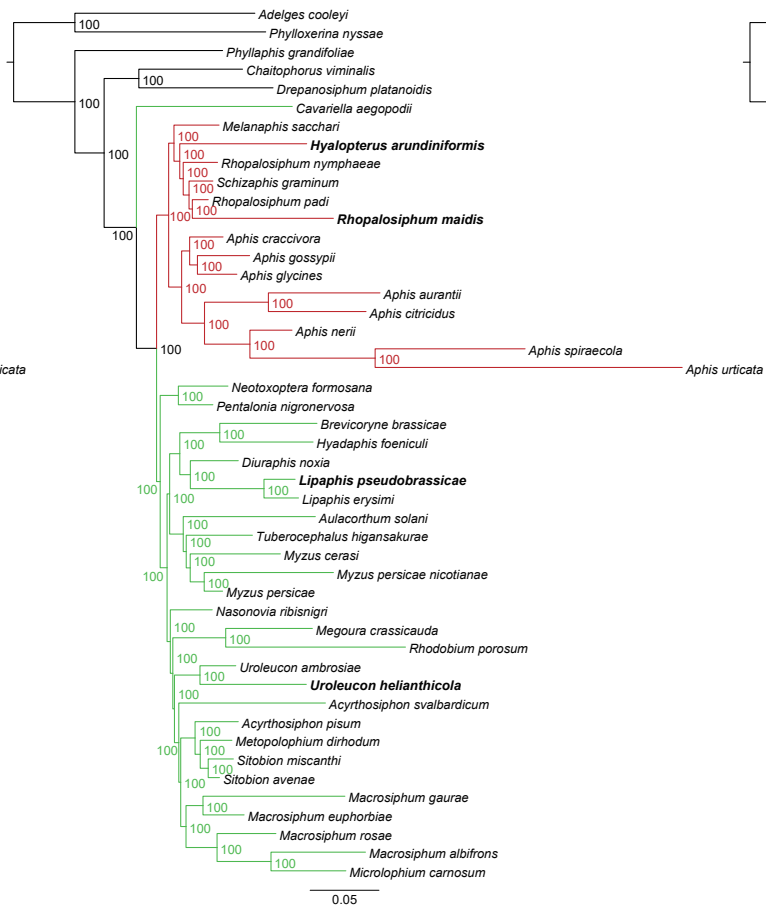

(C) 47taxa\_fna70\_nt123

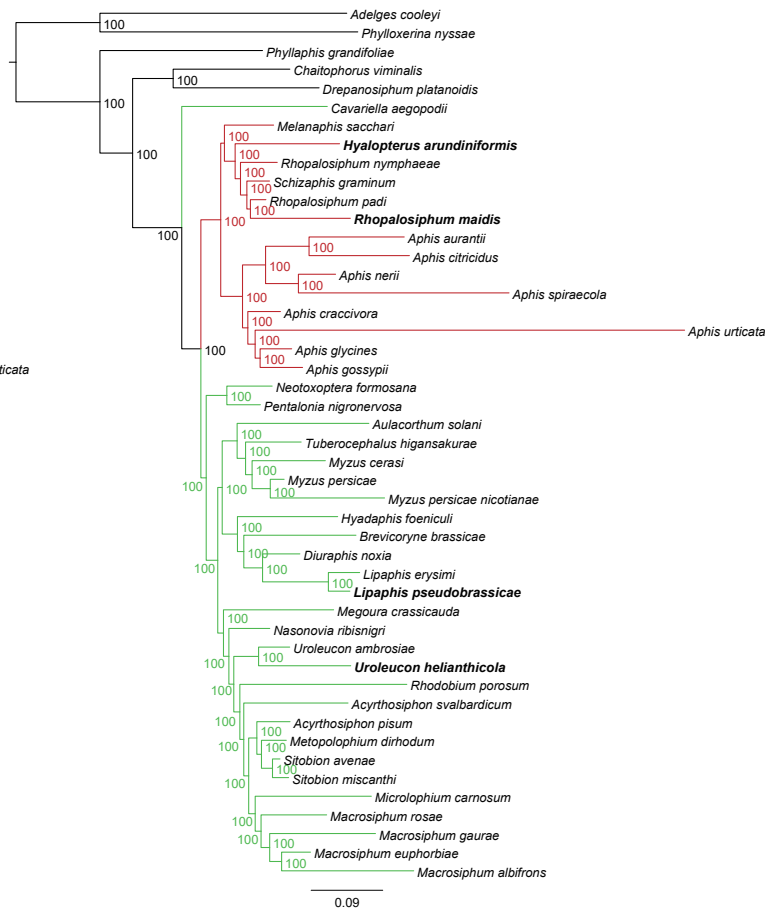

Supplement: Supplementary file 1 [file genes-17-00755-s001.zip › Figure_S12.pdf]

(A) 47taxa\_fna50\_nt12\_wASTRAL

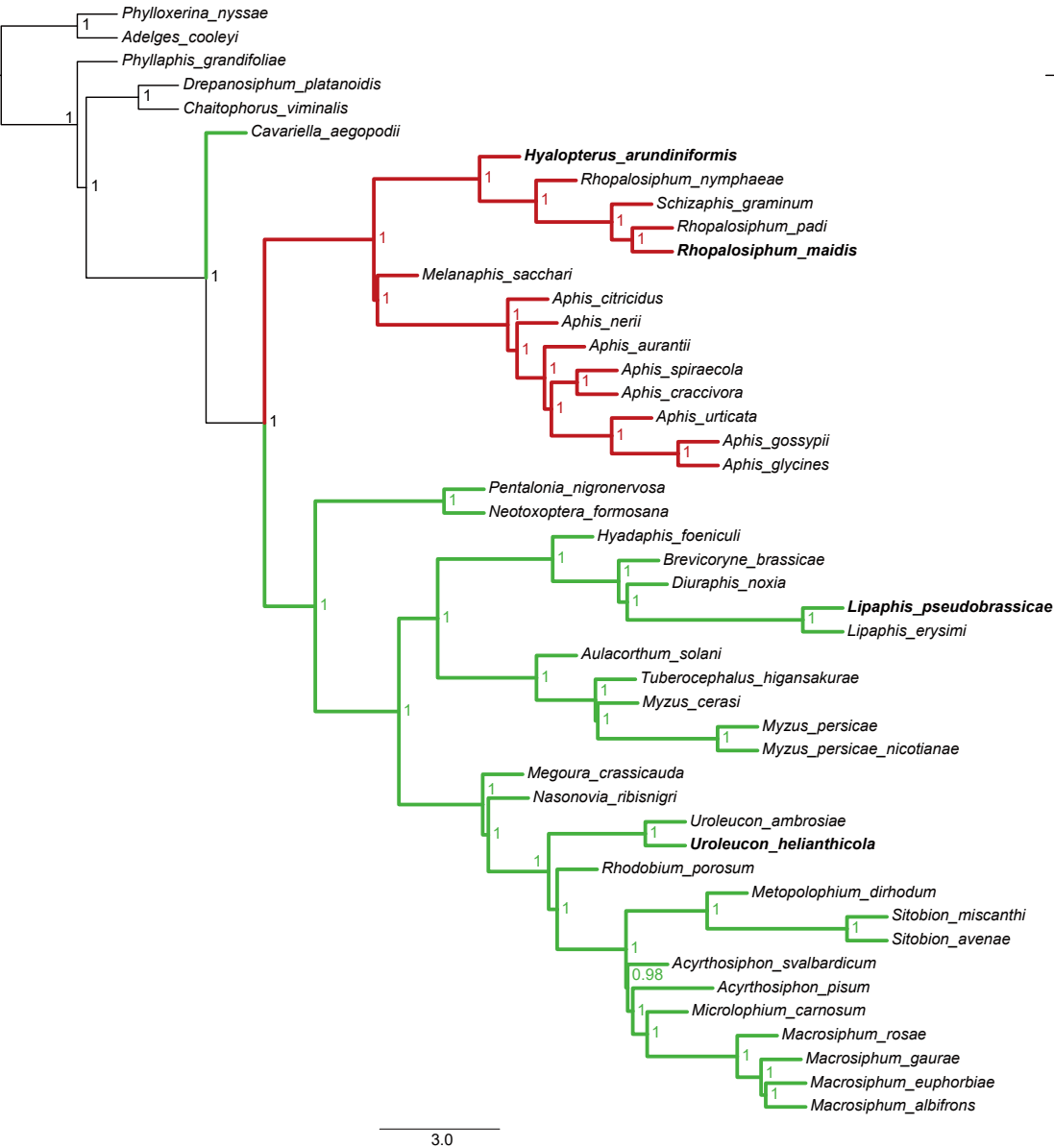

(B) 47taxa\_fna50\_nt123\_wASTRAL

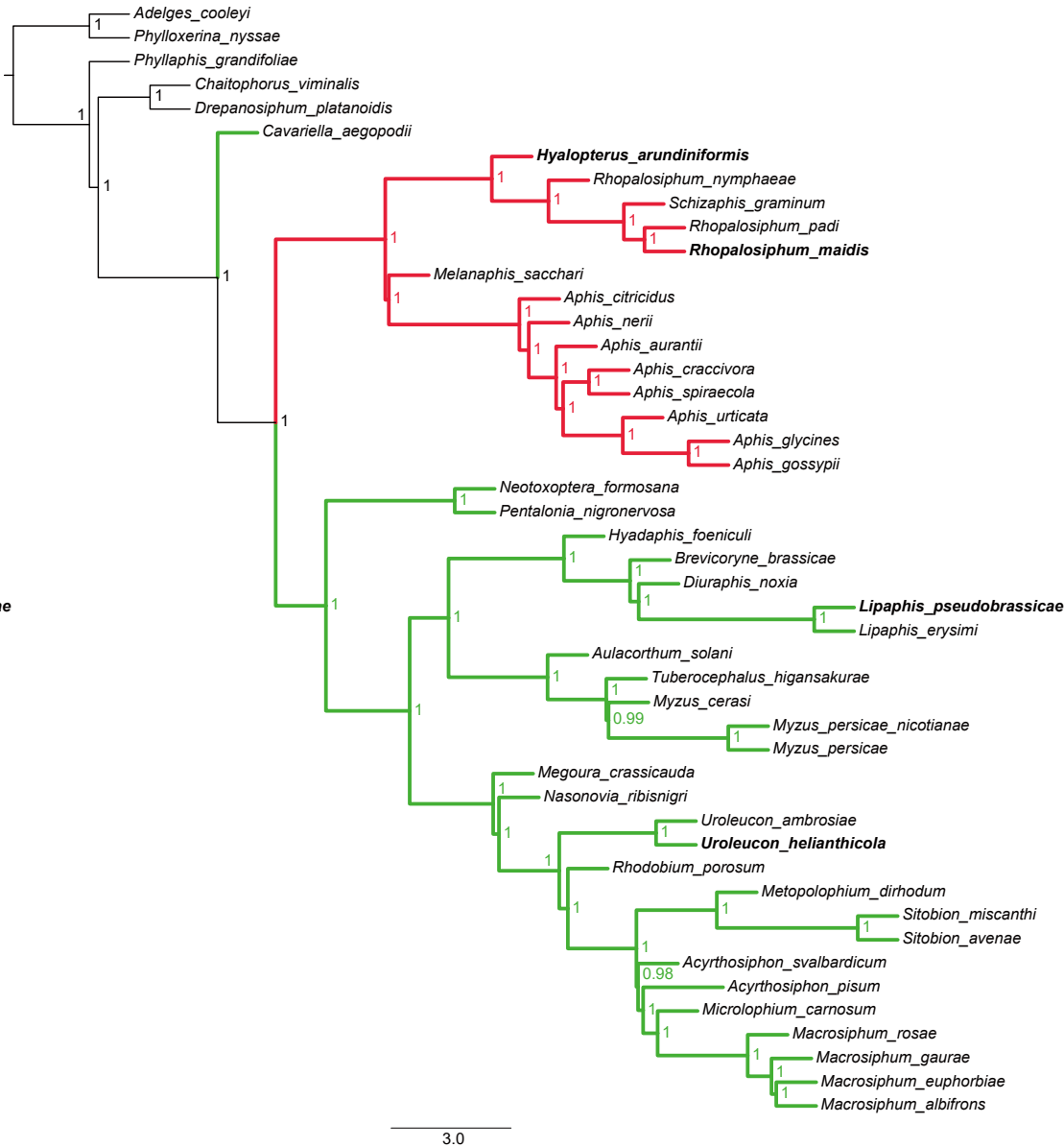

Supplement: Supplementary file 1 [file genes-17-00755-s001.zip › Figure_S13.pdf]

(A) 47taxa\_fna50\_nt12\_consistent\_wASTRAL

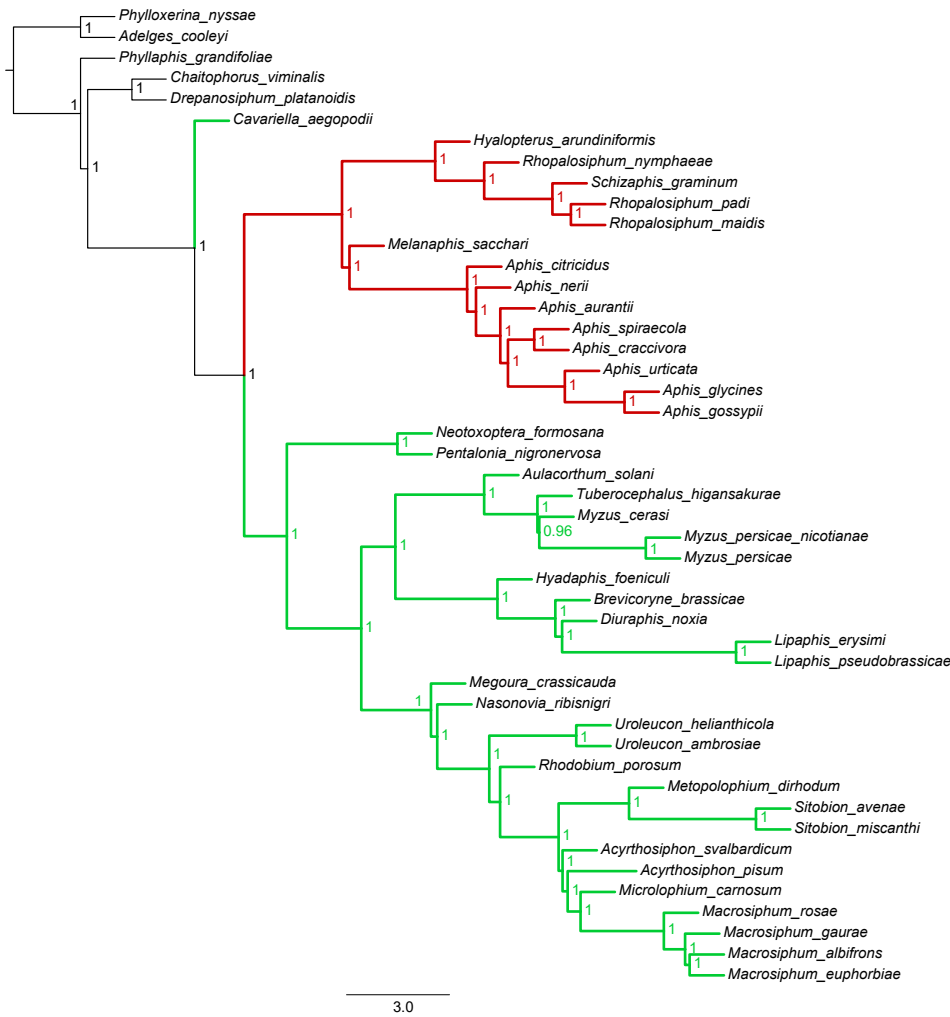

(B) 47taxa\_fna50\_nt12\_consistent\_IQTREE

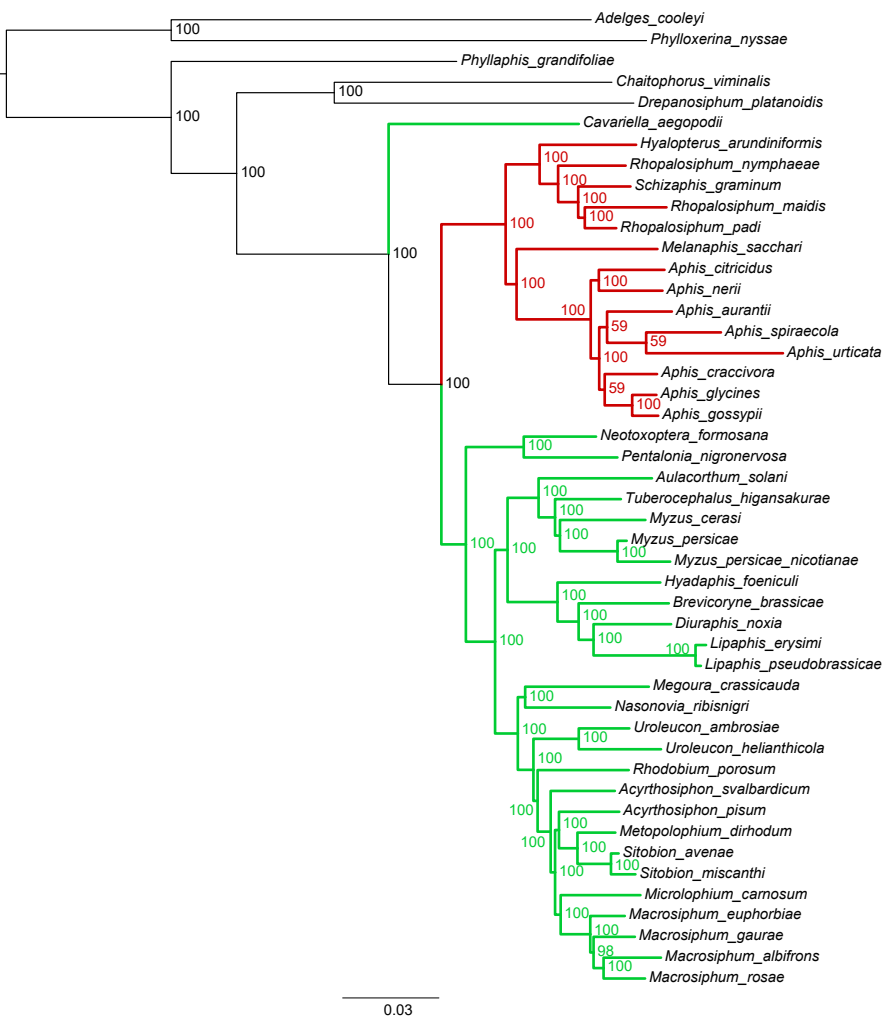

Supplement: Supplementary file 1 [file genes-17-00755-s001.zip › Figure_S14.pdf]

(A) 47taxa\_faa50\_consistent\_wASTRAL

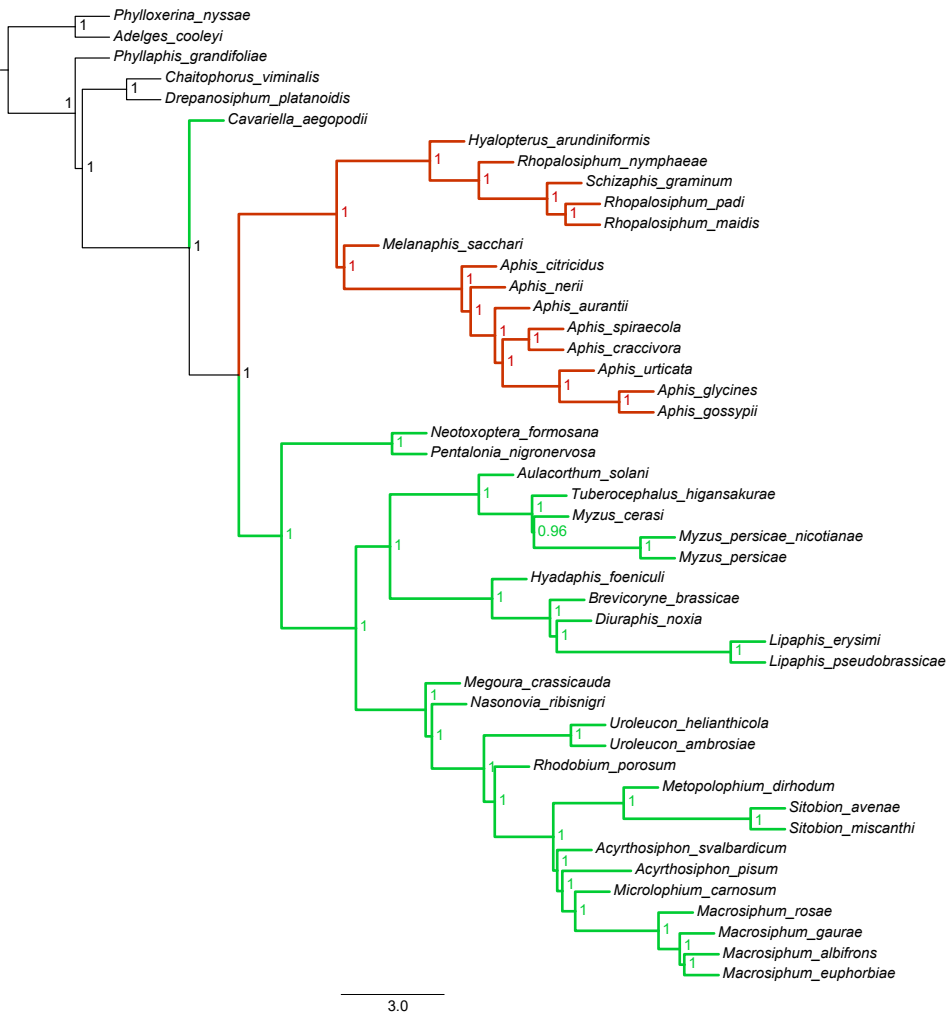

(B) 47taxa\_faa50\_consistent\_IQTREE

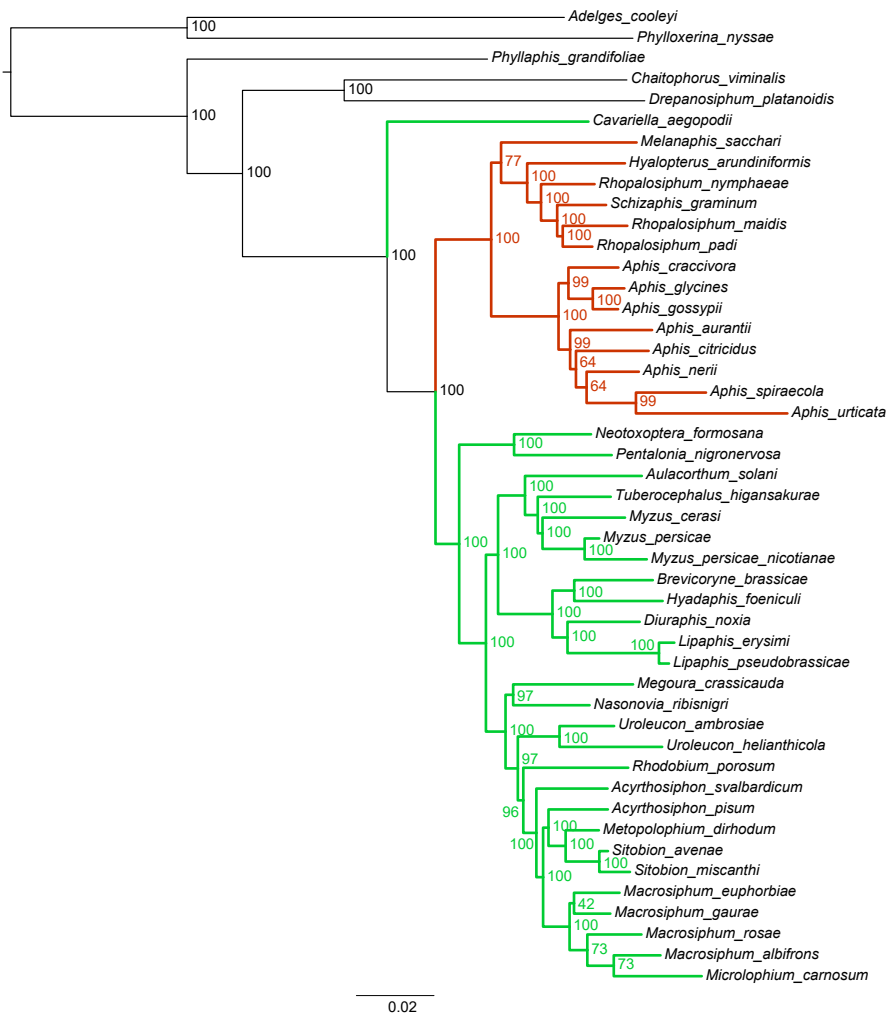

Supplement: Supplementary file 1 [file genes-17-00755-s001.zip › Figure_S15.pdf]

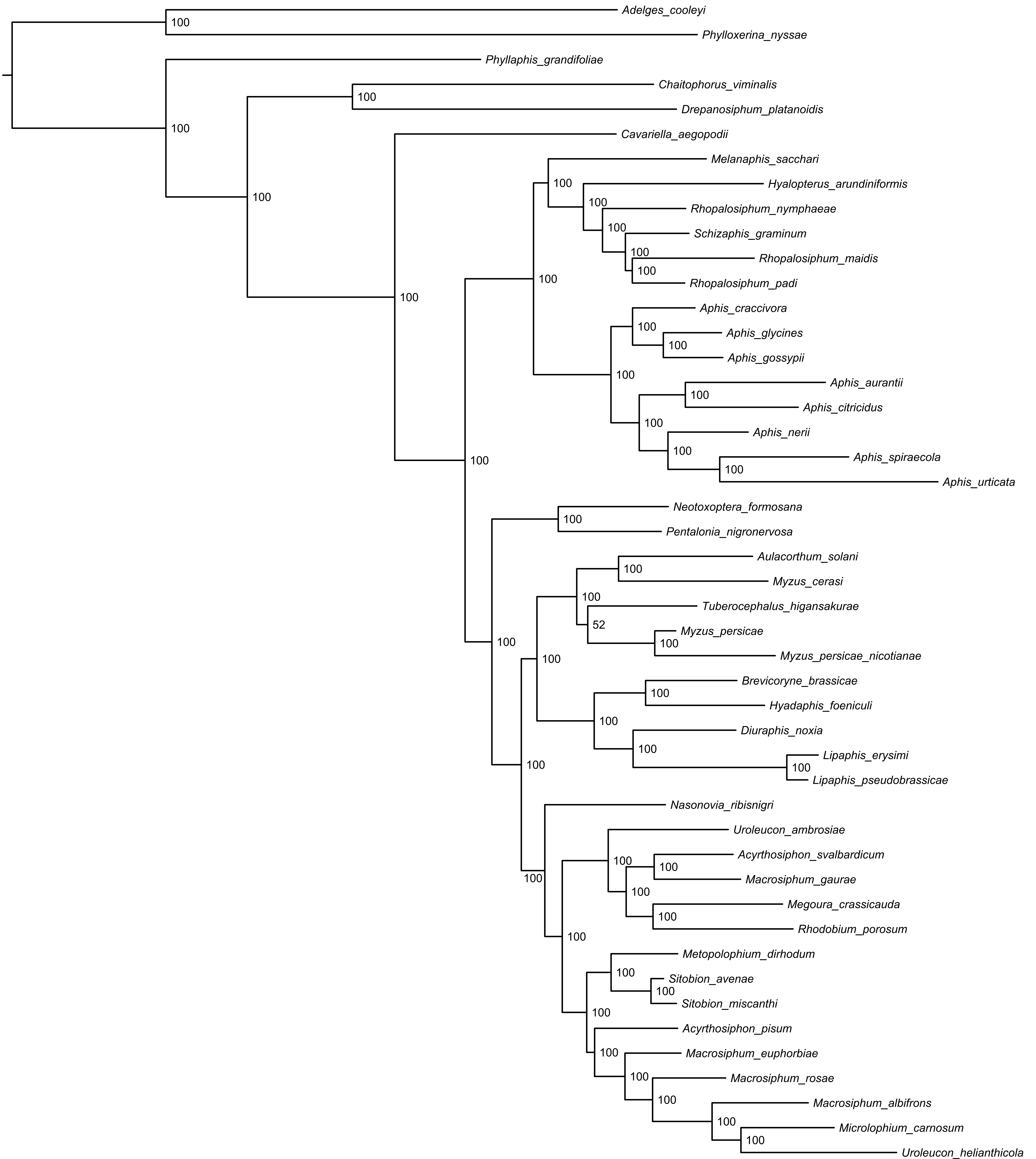

Supplement: Supplementary file 1 [file genes-17-00755-s001.zip › Figure_S16_1-300.pdf]

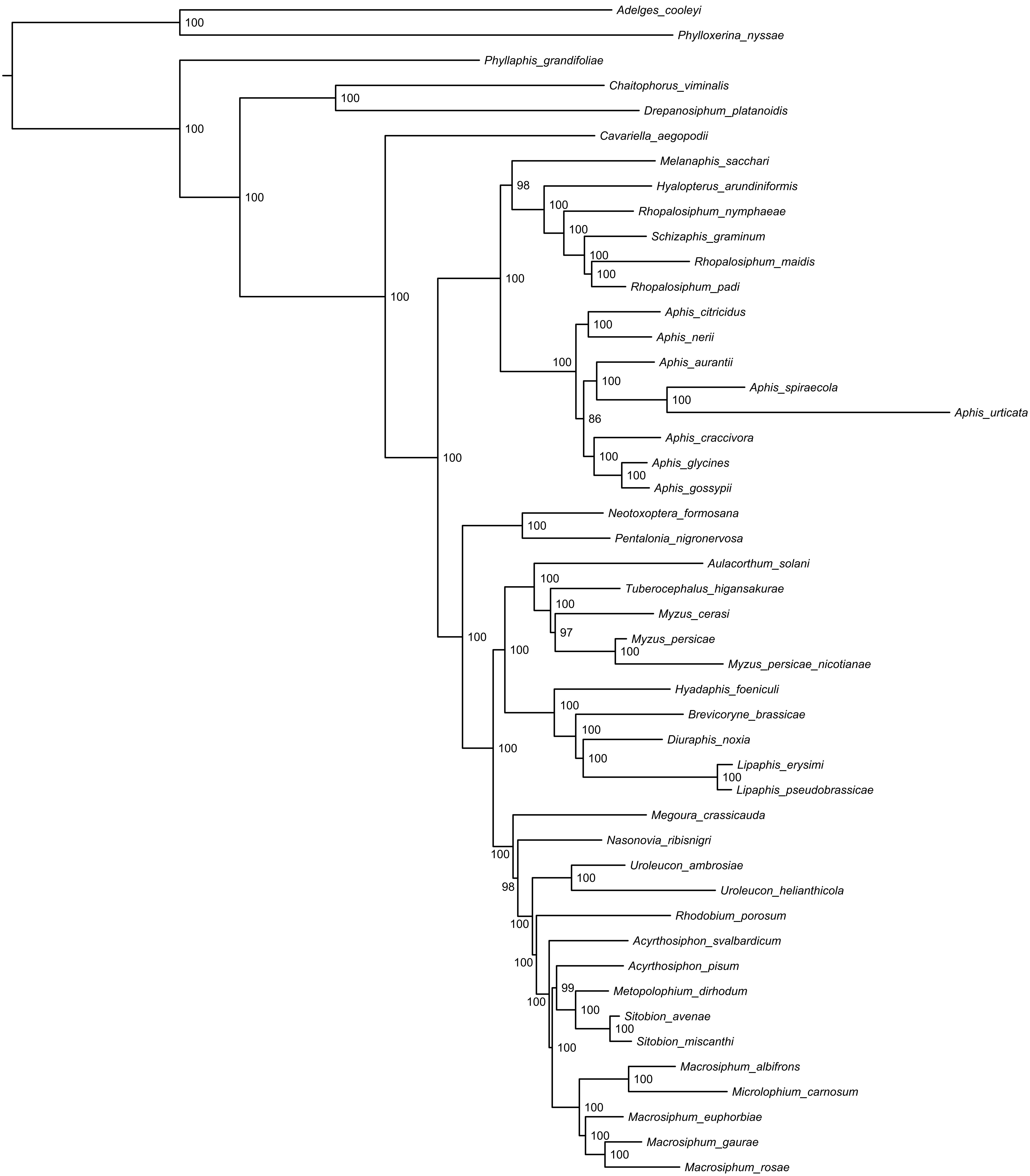

Supplement: Supplementary file 1 [file genes-17-00755-s001.zip › Figure_S17_301-600.pdf]

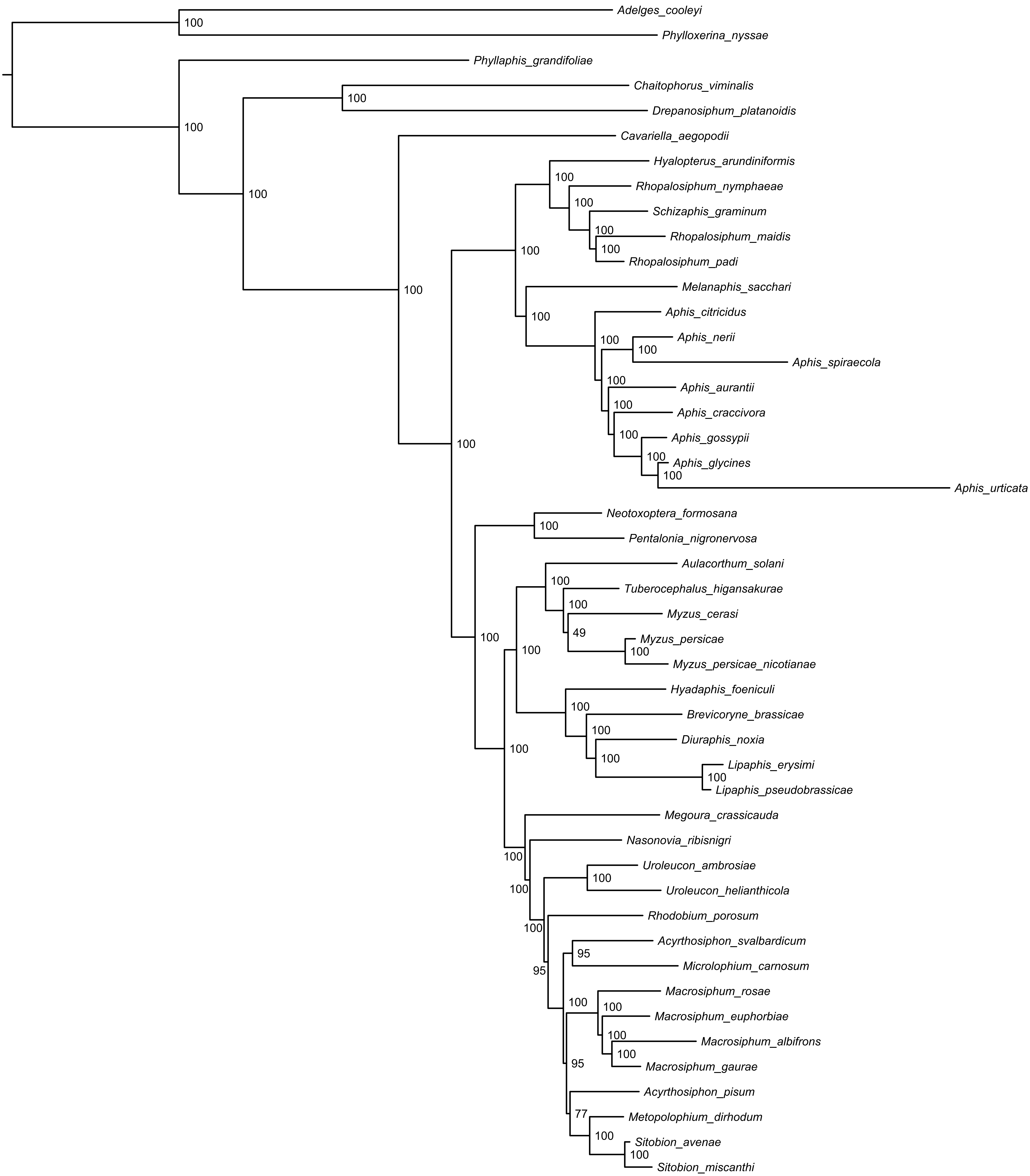

0.04

Supplement: Supplementary file 1 [file genes-17-00755-s001.zip › Figure_S18_601-900.pdf]

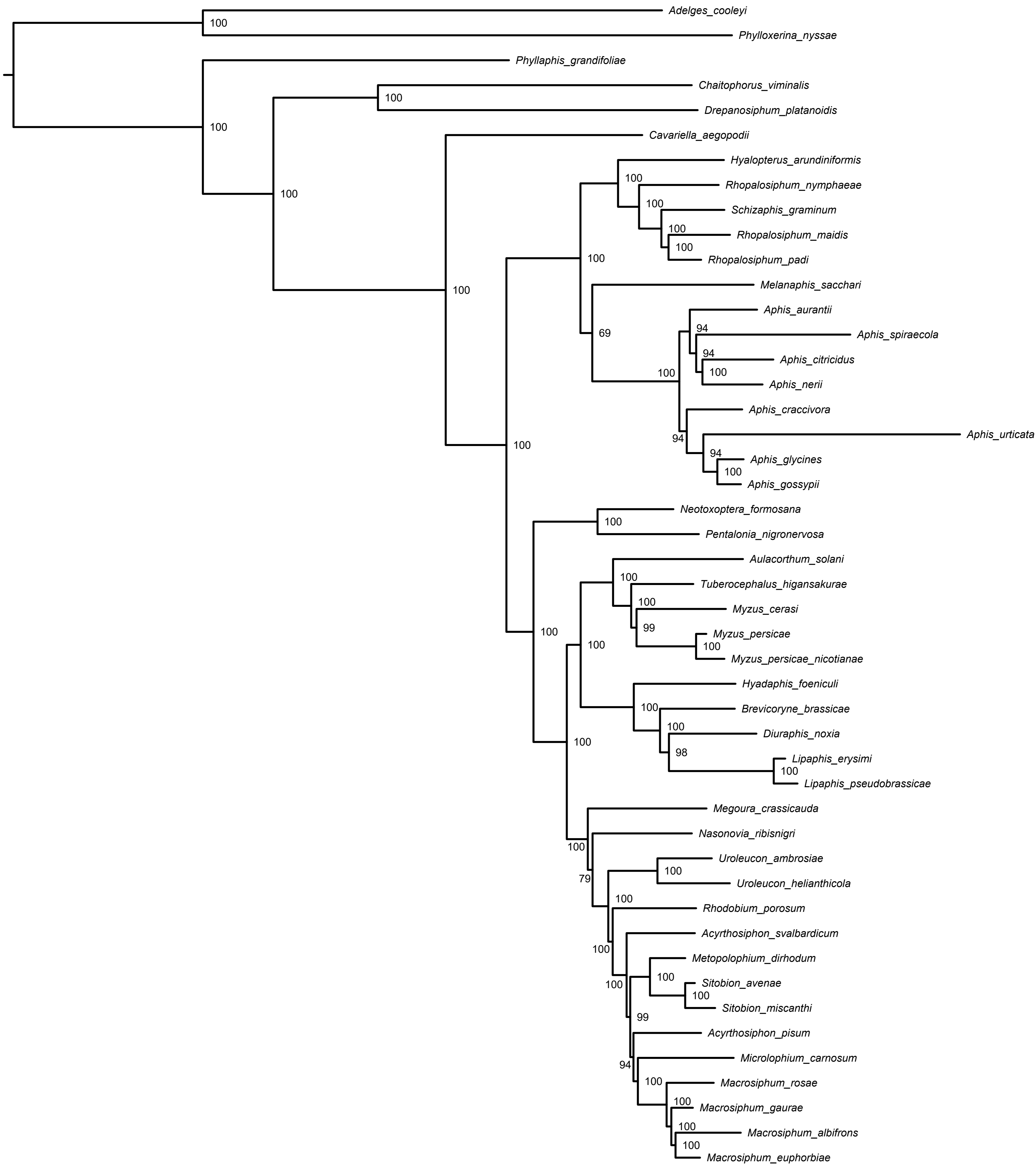

0.04

Supplement: Supplementary file 1 [file genes-17-00755-s001.zip › Figure_S19_901-1200.pdf]

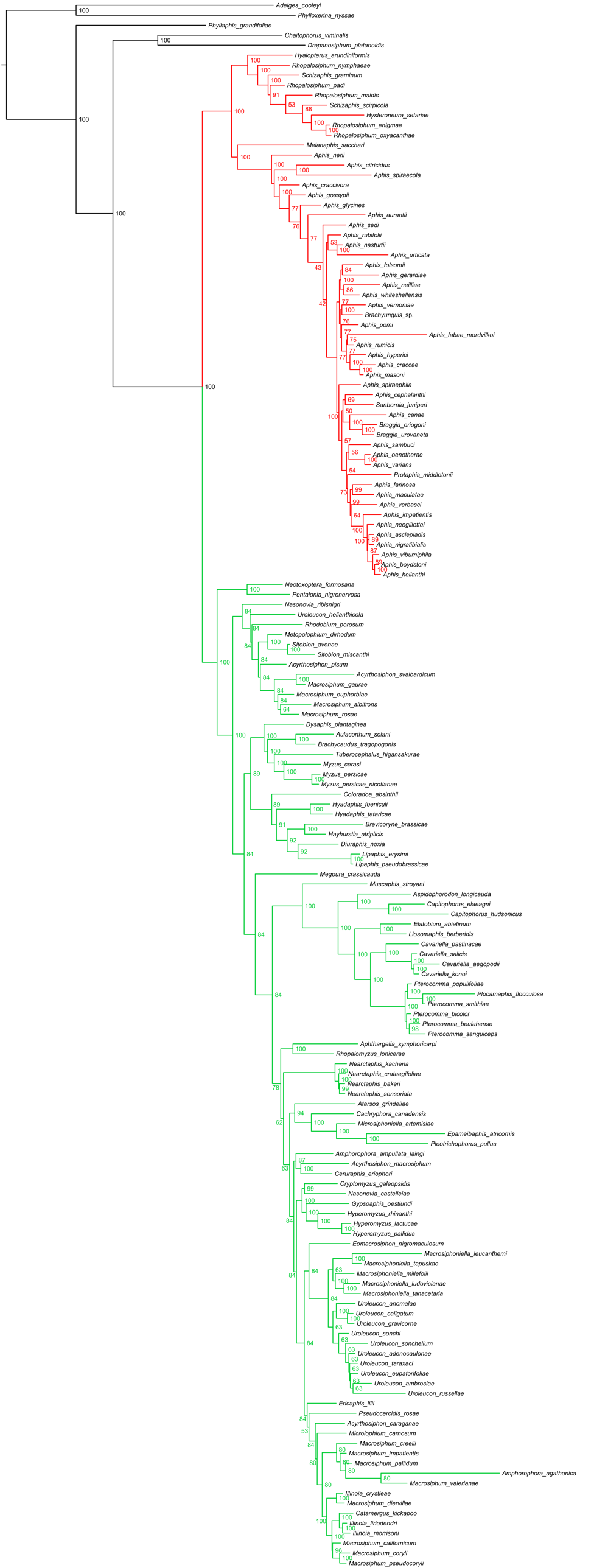

Supplement: Supplementary file 1 [file genes-17-00755-s001.zip › Figure_S2.pdf]

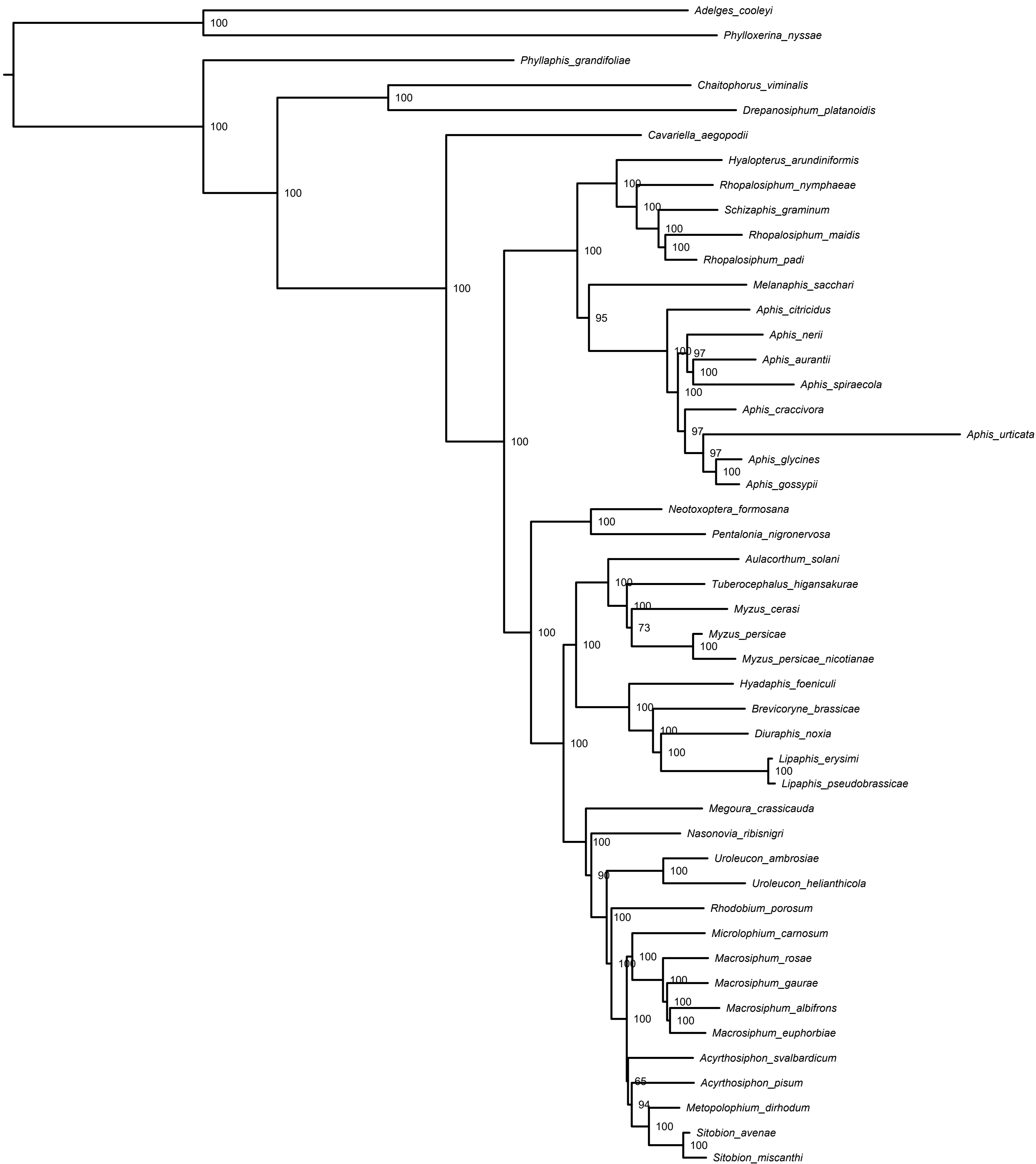

Supplement: Supplementary file 1 [file genes-17-00755-s001.zip › Figure_S20_1201-1500.pdf]

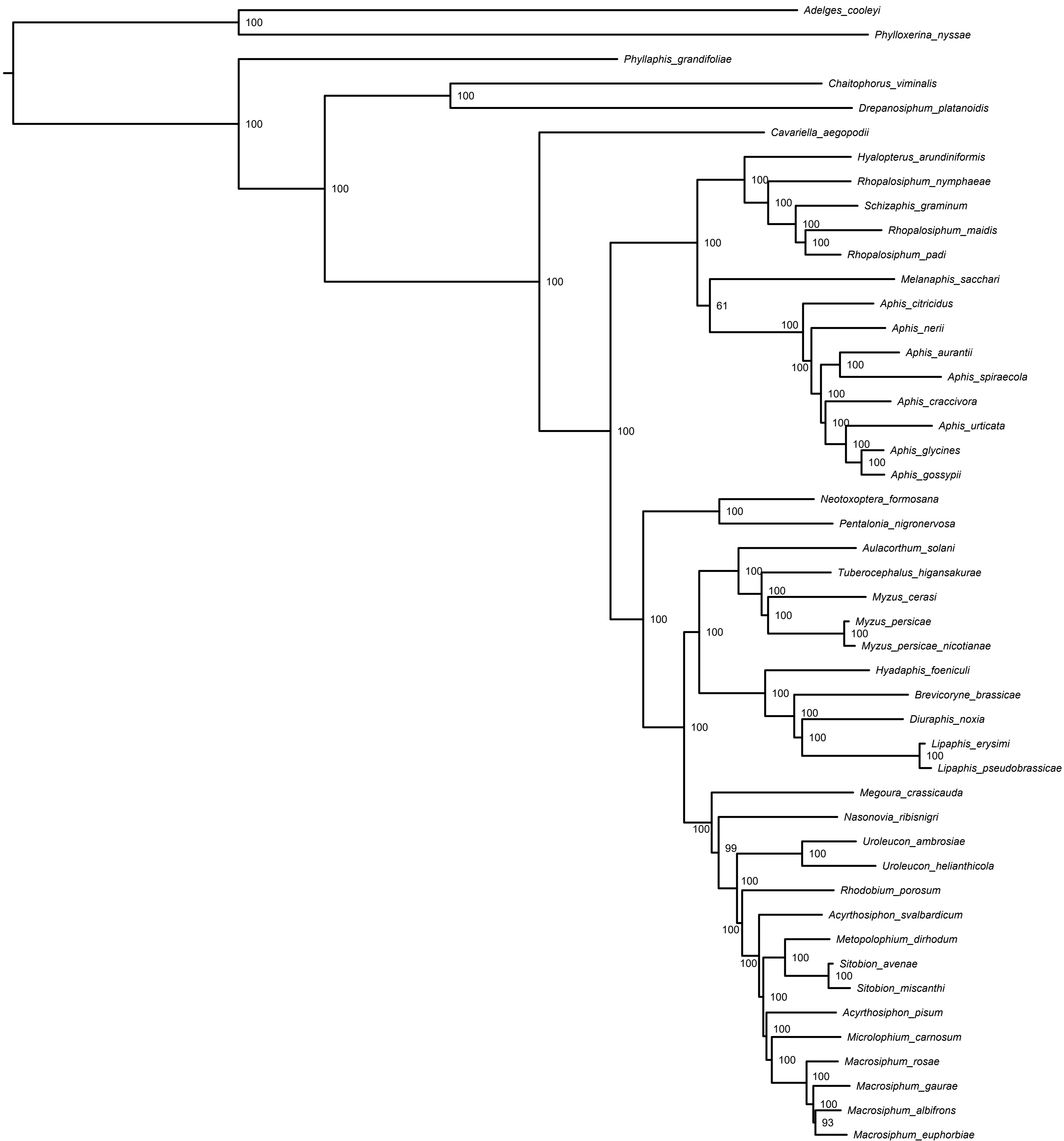

0.04

Supplement: Supplementary file 1 [file genes-17-00755-s001.zip › Figure_S21_1501-1800.pdf]

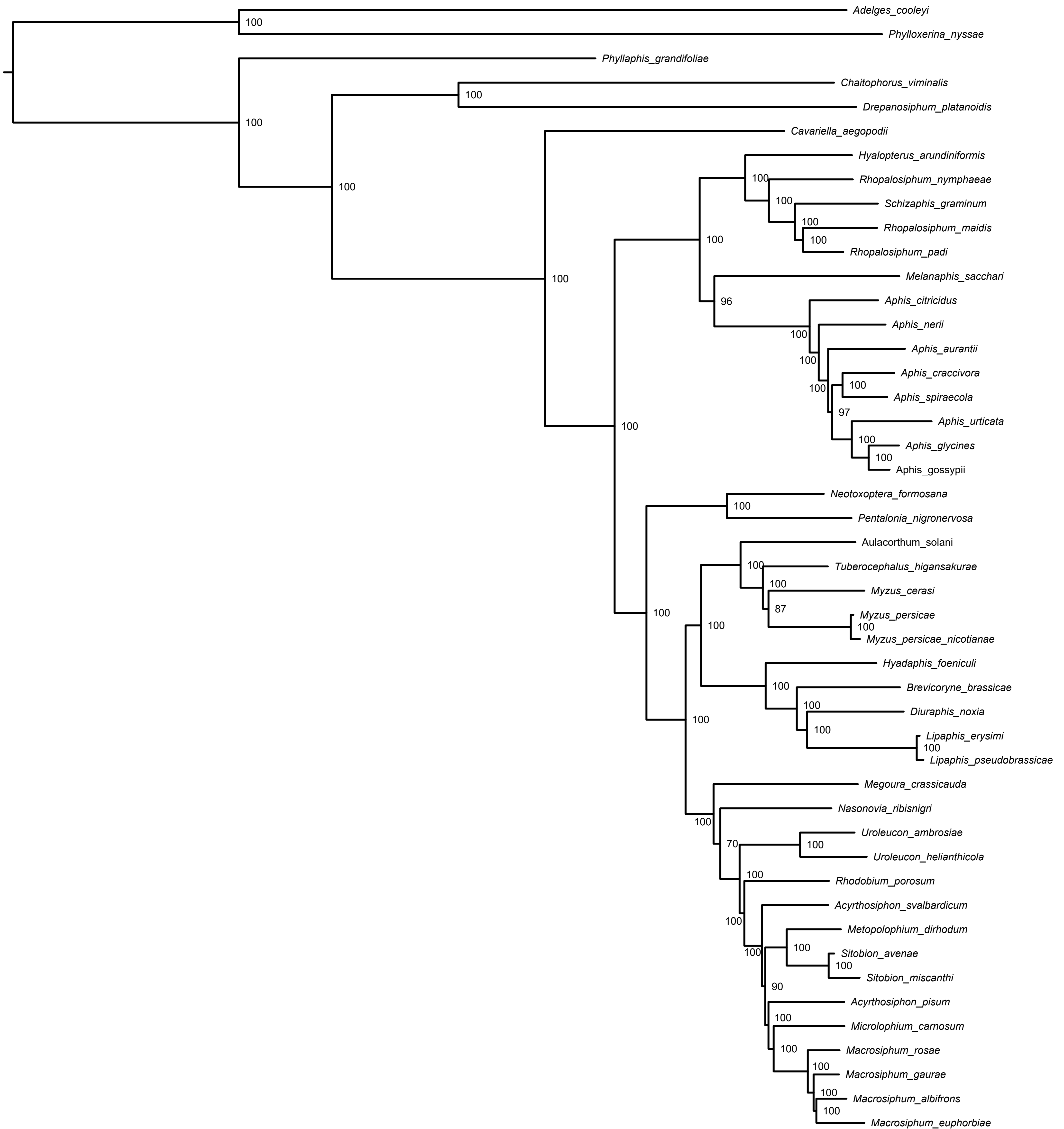

0.04

Supplement: Supplementary file 1 [file genes-17-00755-s001.zip › Figure_S22_1801-2100.pdf]

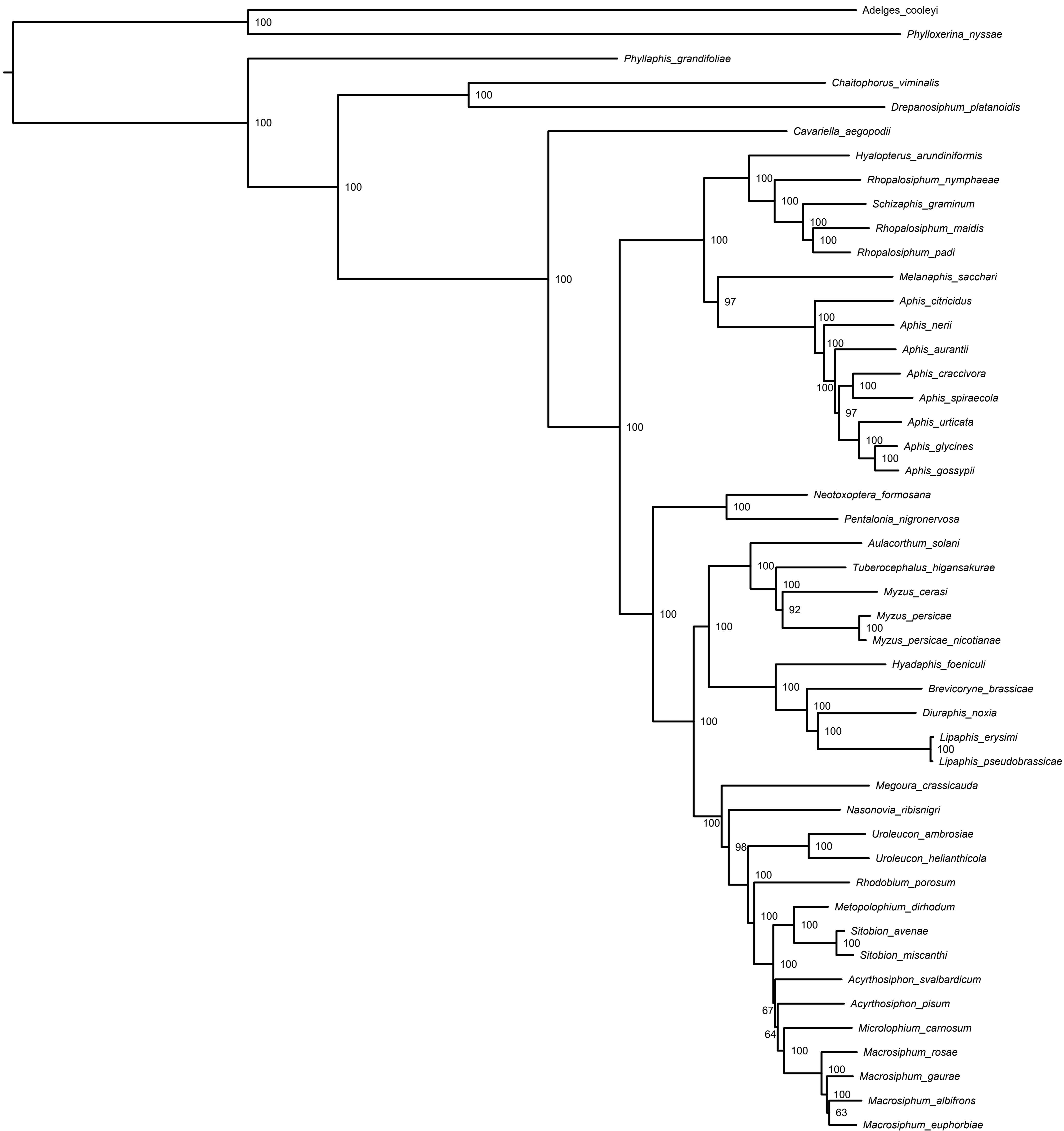

0.04

Supplement: Supplementary file 1 [file genes-17-00755-s001.zip › Figure_S23_2101-2496.pdf]

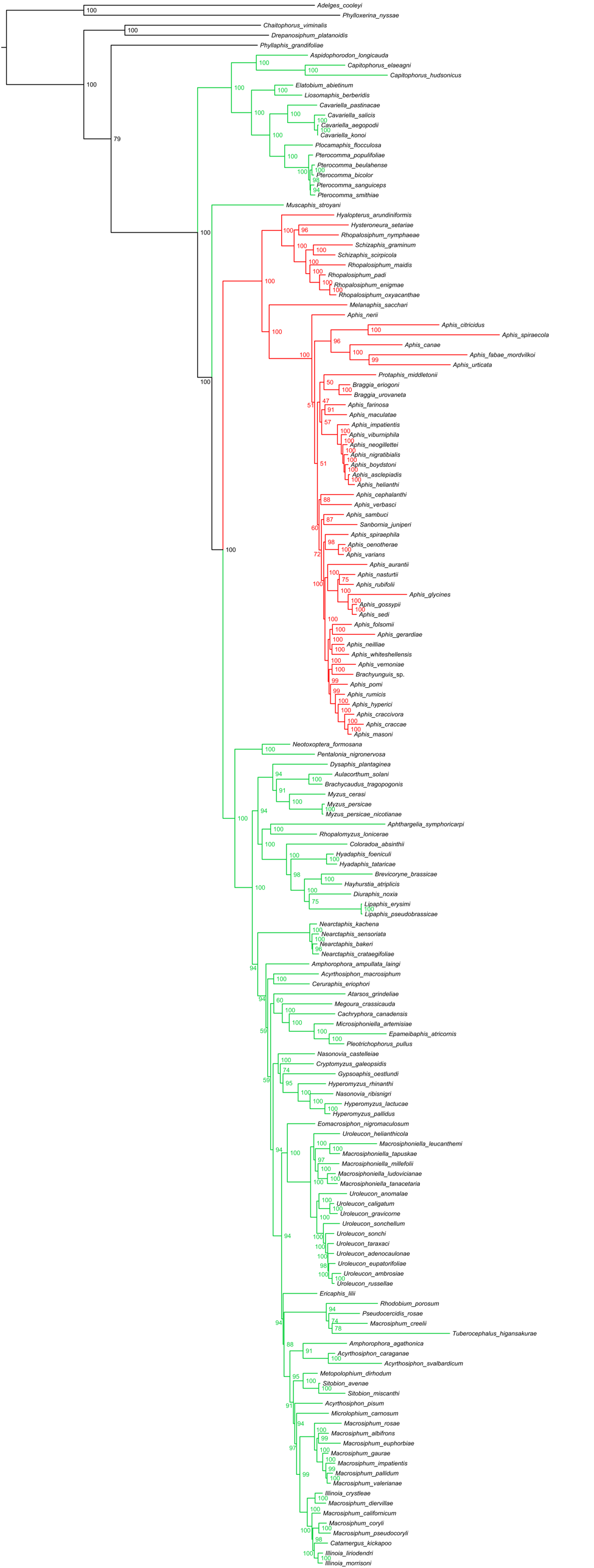

Supplement: Supplementary file 1 [file genes-17-00755-s001.zip › Figure_S3.pdf]

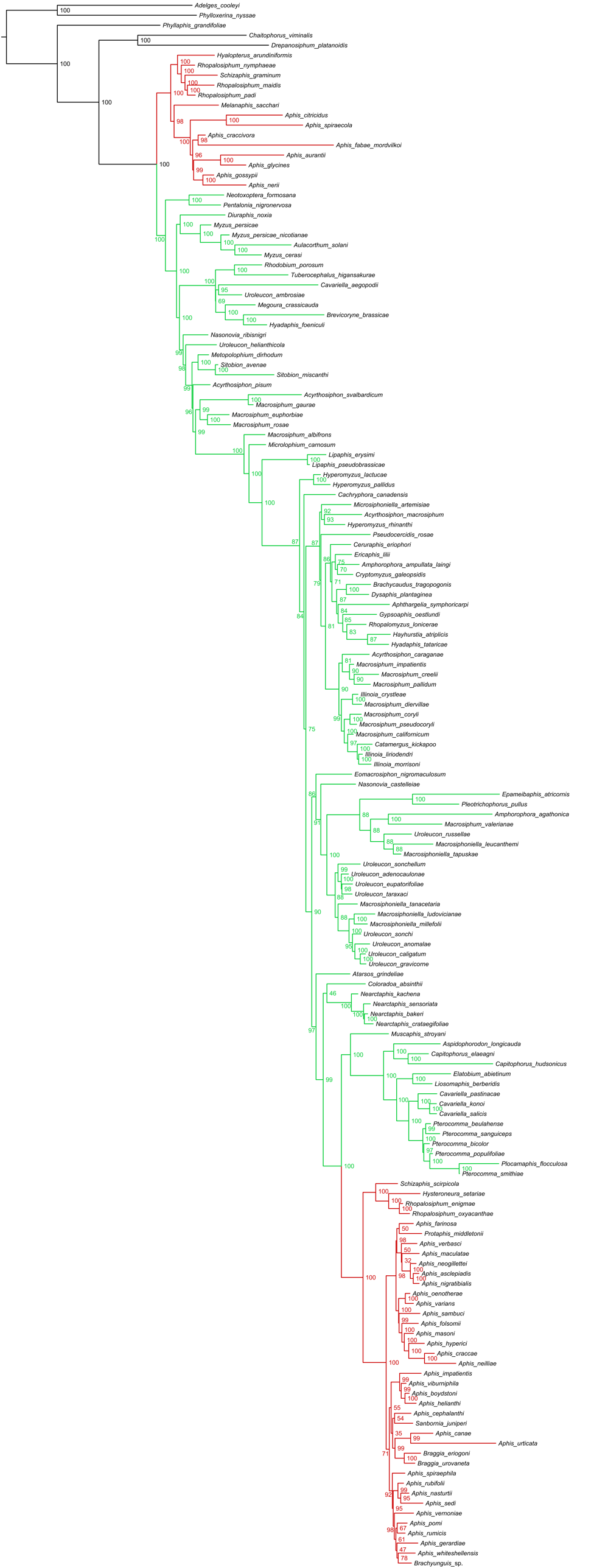

Supplement: Supplementary file 1 [file genes-17-00755-s001.zip › Figure_S4.pdf]

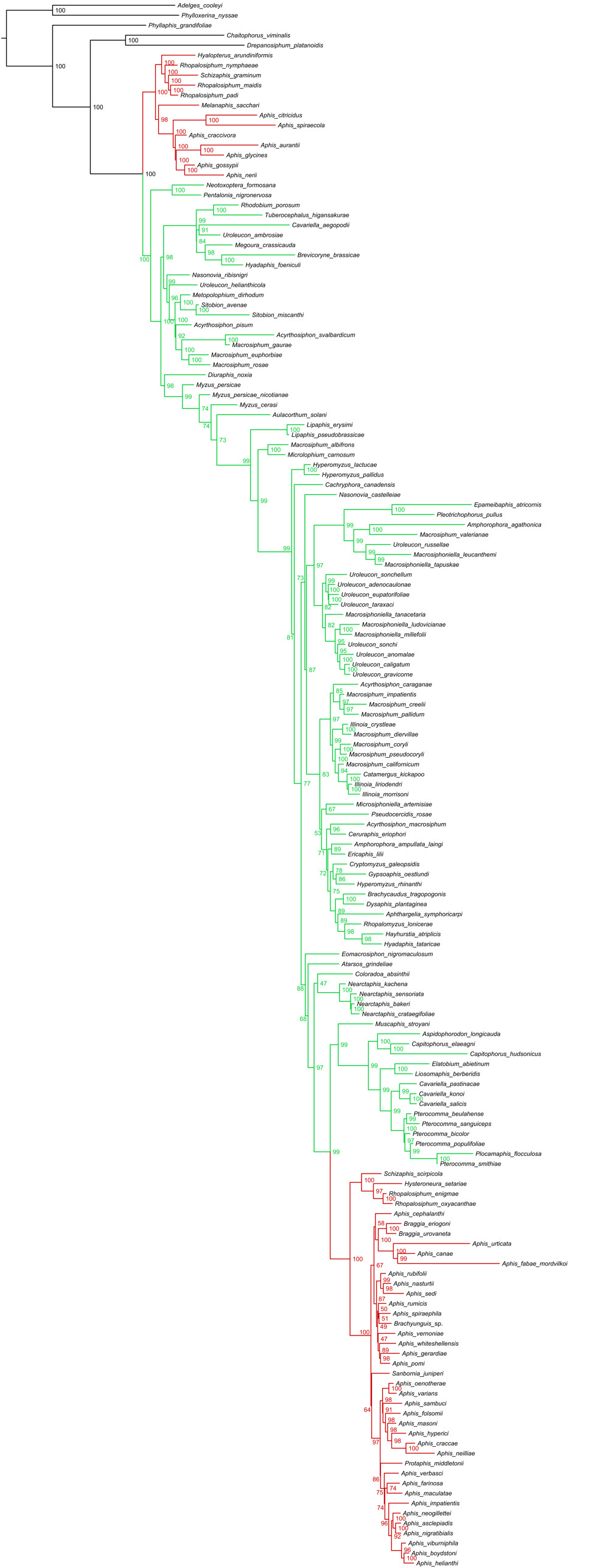

Supplement: Supplementary file 1 [file genes-17-00755-s001.zip › Figure_S5.pdf]

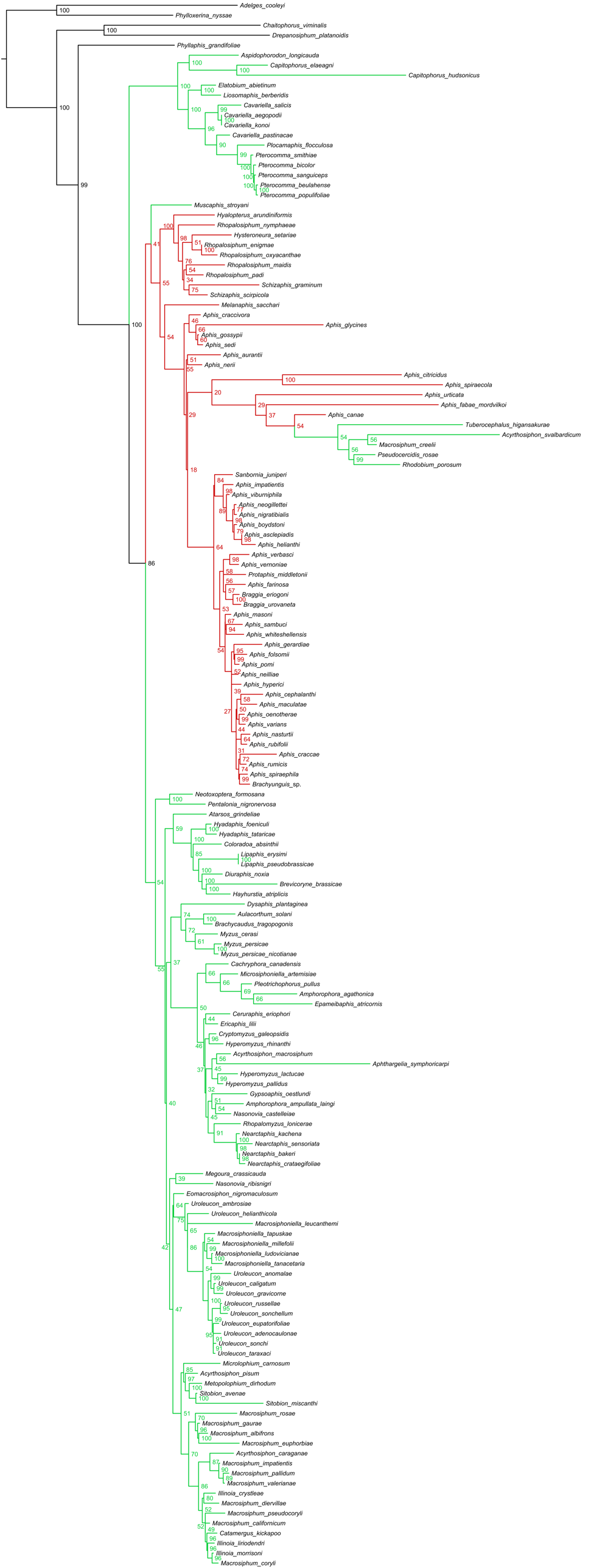

Supplement: Supplementary file 1 [file genes-17-00755-s001.zip › Figure_S6.pdf]

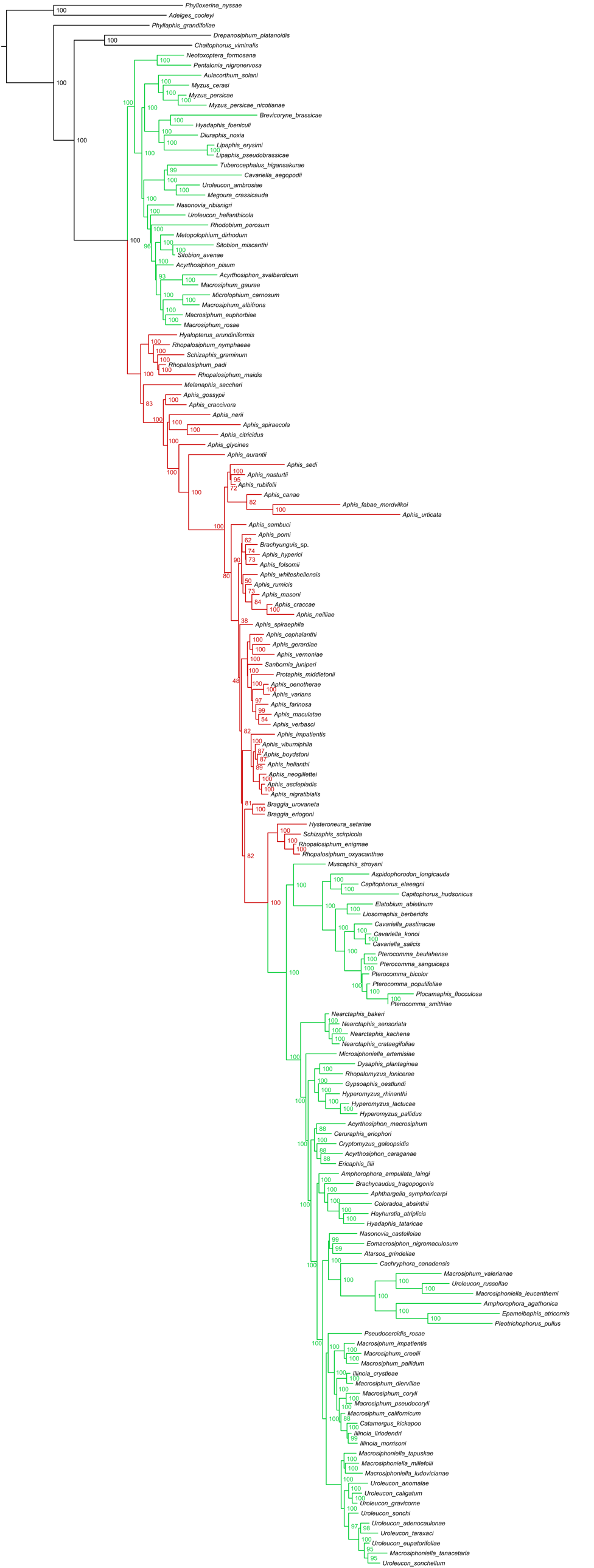

0.02

Supplement: Supplementary file 1 [file genes-17-00755-s001.zip › Figure_S7.pdf]

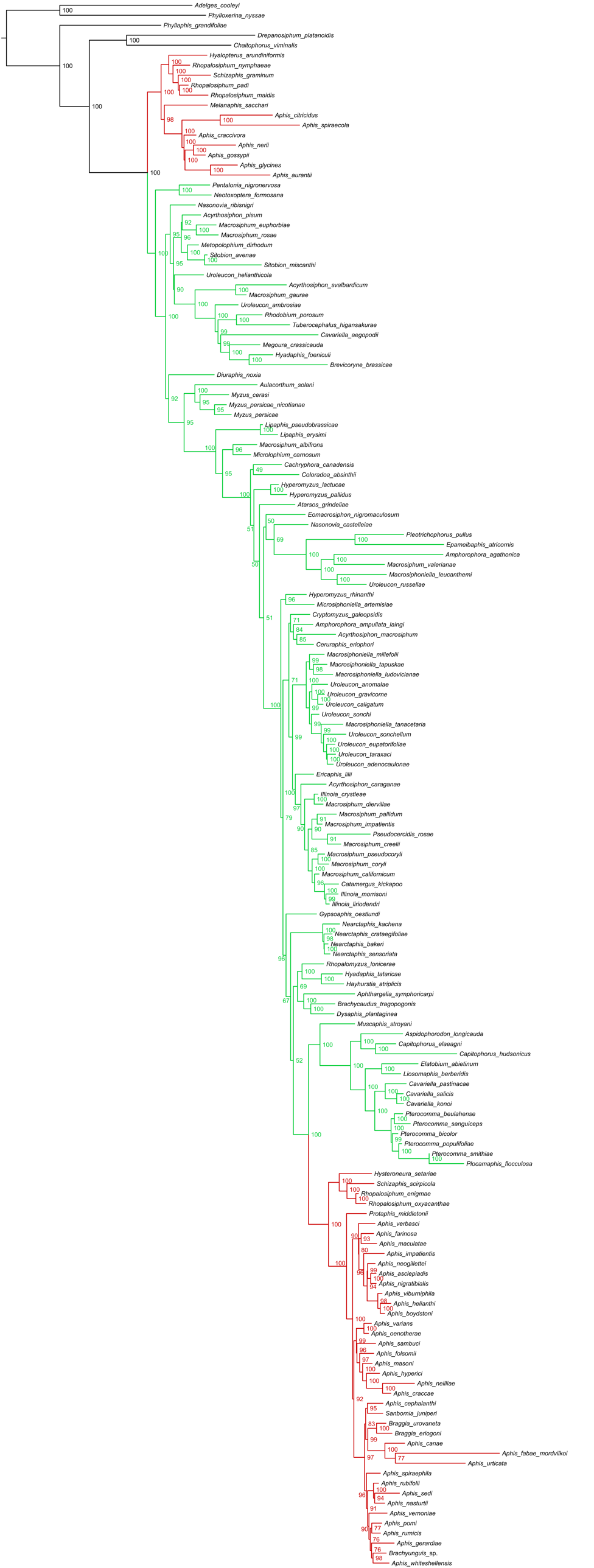

Supplement: Supplementary file 1 [file genes-17-00755-s001.zip › Figure_S8.pdf]

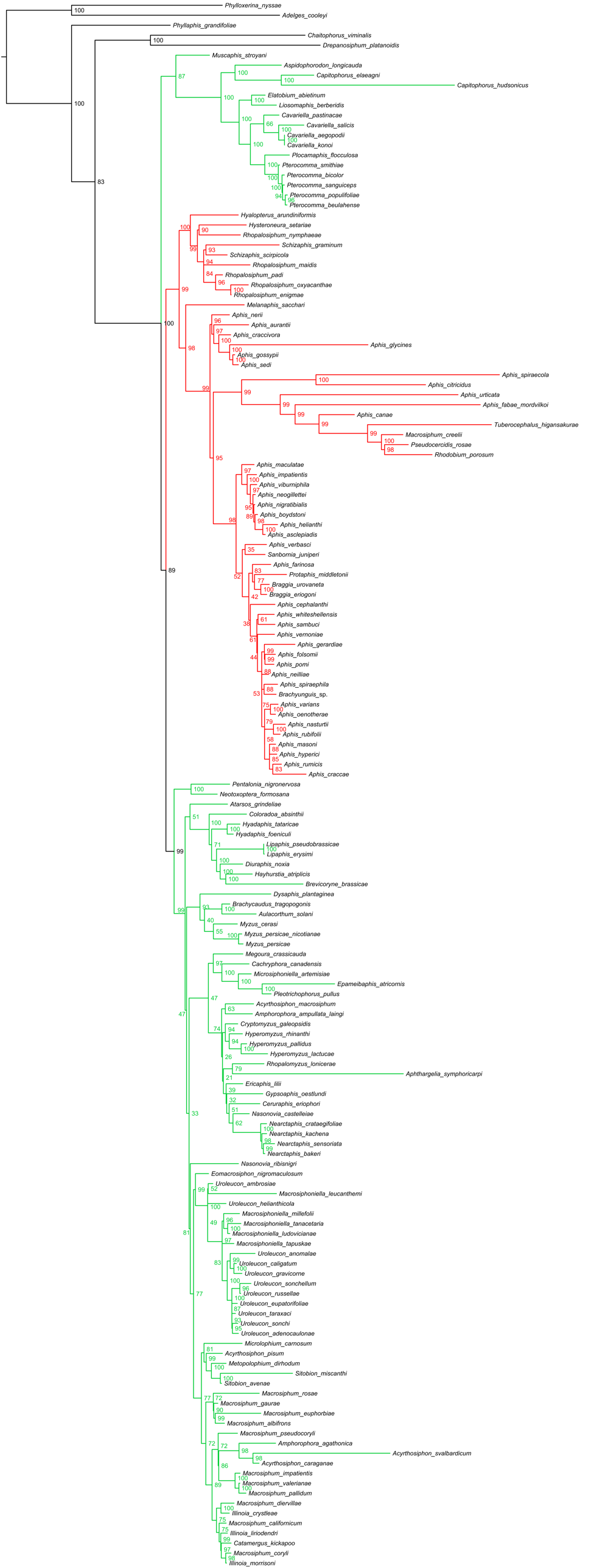

Supplement: Supplementary file 1 [file genes-17-00755-s001.zip › Figure_S9.pdf]
